# Development of a scoring function for comparing simulated and experimental tumor spheroids

**DOI:** 10.1371/journal.pcbi.1010471

**Published:** 2023-03-30

**Authors:** Julian Herold, Eric Behle, Jakob Rosenbauer, Jacopo Ferruzzi, Alexander Schug

**Affiliations:** 1 HIDSS4Health - Helmholtz Information and Data Science School for Health, Karlsruhe/Heidelberg, Germany; 2 Steinbuch Centre for Computing, Karlsruhe Institute of Technology, Karlsruhe, Germany; 3 NIC Research Group Computational Structural Biology, Jülich Research Center, Jülich, Germany; 4 Department of Bioengineering, University of Texas at Dallas, Richardson, Texas, United States of America; Max Planck Institute for Evolutionary Biology: Max-Planck-Institut fur Evolutionsbiologie, GERMANY

## Abstract

Progress continues in the field of cancer biology, yet much remains to be unveiled regarding the mechanisms of cancer invasion. In particular, complex biophysical mechanisms enable a tumor to remodel the surrounding extracellular matrix (ECM), allowing cells to invade alone or collectively. Tumor spheroids cultured in collagen represent a simplified, reproducible 3D model system, which is sufficiently complex to recapitulate the evolving organization of cells and interaction with the ECM that occur during invasion. Recent experimental approaches enable high resolution imaging and quantification of the internal structure of invading tumor spheroids. Concurrently, computational modeling enables simulations of complex multicellular aggregates based on first principles. The comparison between real and simulated spheroids represents a way to fully exploit both data sources, but remains a challenge. We hypothesize that comparing any two spheroids requires first the extraction of basic features from the raw data, and second the definition of key metrics to match such features. Here, we present a novel method to compare spatial features of spheroids in 3D. To do so, we define and extract features from spheroid point cloud data, which we simulated using Cells in Silico (CiS), a high-performance framework for large-scale tissue modeling previously developed by us. We then define metrics to compare features between individual spheroids, and combine all metrics into an overall deviation score. Finally, we use our features to compare experimental data on invading spheroids in increasing collagen densities. We propose that our approach represents the basis for defining improved metrics to compare large 3D data sets. Moving forward, this approach will enable the detailed analysis of spheroids of any origin, one application of which is informing *in silico* spheroids based on their *in vitro* counterparts. This will enable both basic and applied researchers to close the loop between modeling and experiments in cancer research.

## Author summary

Cells within a tumor use various methods to escape and thereby invade into healthy parts of the body. These methods are studied experimentally by examining tumor spheroids, spherical aggregates of hundreds to thousands of individual cells. Such spheroids can also be simulated, and the comparison of both simulations and experiments is desirable. Here, we present an analysis strategy for the comparison of tumor spheroids of any origin. Using this strategy, we aim to increase the information gained from the data and improve the collaborative potential between experimentalists and theorists.

This is a *PLOS Computational Biology* Methods paper.

## 1 Introduction

**Fig 1:**
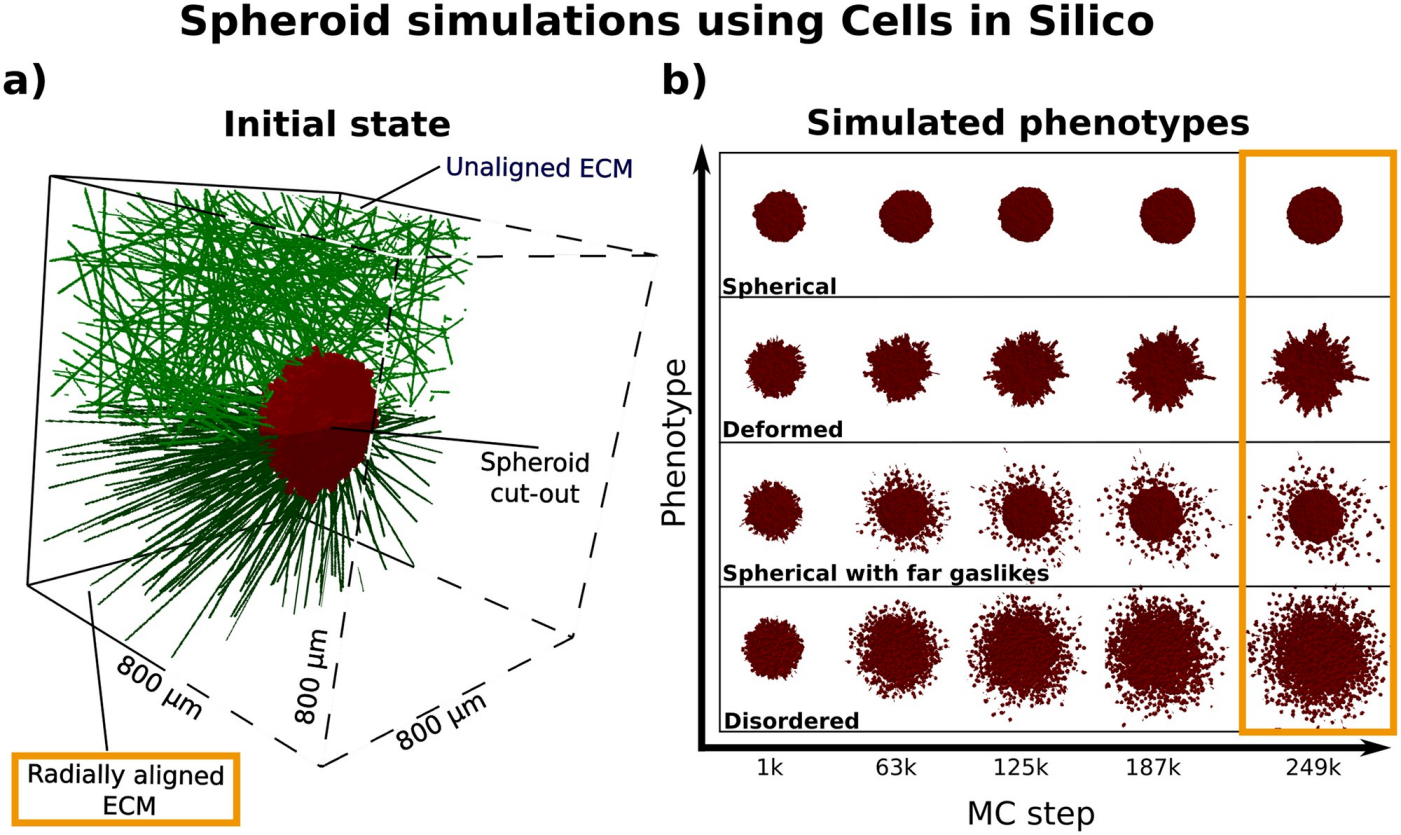
Simulated spheroids and emerging phenotypes. **a)** Cut-outs of the initial states of simulated spheroids for two ECM alignments. Each spheroid, shown in red and containing roughly 2000 cells, was placed into an (800 μm)^3^ volume and surrounded by either an unaligned or radially aligned ECM (green fibers). To improve visibility, the front half of the volume (dashed lines) is not shown **b)** Time evolution of simulated spheroids displaying four different phenotypes: “spherical”, “deformed”, “spherical with far gaslikes”, “disordered”. The ECM is radially aligned for these phenotypes, and is not shown in order to highlight the spheroid morphology. Each phenotype resulted from different combinations of parameters connected to the cell motility, the cell-cell adhesion and the interaction with the ECM (see Section 2.1 and Table 1). Each simulation lasted 250 000 Monte-Carlo (MC) steps, and shown are five snapshots for each simulation. A single MC step corresponds to roughly 1 s of real time in the context of this study. Throughout our investigation, we focused on the final configuration (orange rectangle), and used five replicates from each phenotype.

The worldwide challenge of fighting cancer is as urgent as ever [1, 2]. When trying to understand the mechanisms driving the disease, one is faced with a complex and wildly inhomogeneous landscape of cellular properties and interactions, which vary both within and between cancer types [3–5]. Furthermore, cancer is not one single disease, but rather refers to a large number of diseases with shared characteristics, which are captured in the hallmarks of cancer [2, 6]. The processes underlying these diseases, such as the rise of malignancy via loss of cell-cell adhesion and subsequent increased motility [7], span a wide range of scales, both in space and in time [8]. To further the understanding of cancer, it is crucial to decipher how these processes interact and lead to the formation of macroscopic invasive tumors. Thus, combating cancer requires input from many different domains of science, such as biology, medicine, and pharmacology, but also physics, computer science, and mathematics [8, 9]. Unfortunately, time-resolved analysis of *in vivo* tumor tissue is challenging, as due to low spatial or temporal resolution of imaging methods, single-cell resolution 4D trajectories are not yet widely applicable. To increase accessibility for analysis, the system has to be divided into smaller subsystems. Thus, *in vitro* and *in silico* models are created, allowing the study of individual aspects of the system. An *in vitro* example is the study of tumor spheroids, which represent a useful model system for studying tumor growth and cell dynamics [10]. Tumor spheroids are spherical arrangements of hundreds to thousands of cells, which can be placed within a structural extracellular matrix (ECM), e.g. a collagen scaffold. They are widely used for studying e.g. drug response, tissue fluidity and tumor invasion [11–13]. On the *in silico* side, tumor growth models of varying degree of coarse-graining are being developed [14–16], some of which are also applied to simulate tumor spheroids [17, 18]. Thus, both experimentalists and theorists generate data for the same systems, but these studies are often not compared quantitatively. Quantitative comparison is an important step towards fully leveraging the results of both groups, and in this context requires an adaptive and robust comparison strategy for spheroid data, regardless of its origin. In prior studies, Browning et al. have investigated structural aspects of melanoma-derived spheroids by building a data-analysis pipeline for spheroid images resulting from confocal microscopy [19], and Szymańska et al. have studied the proliferation behavior of spheroids using Bayesian inference [20]. However, to our knowledge there is currently no strategy for systematically comparing 3D structural data between two spheroids, which can be obtained *in vitro* using stacked multiphoton microscopy images [13]. Hence, in this study, we want to provide a toolbox of features which may be extracted from a given 3D structure of a spheroid, and metrics to compare these extracted features between different spheroids. These features and metrics can be used on their own, or in combination, to obtain an overall deviation score. Our strategy utilizes point cloud data, in which each point denotes the position of a cell. Importantly, this enables the comparison of both simulated and experimental spheroids. To demonstrate this, we applied our toolbox to previously published data that captured structural differences in triple-negative breast cancer spheroids invading into a collagenous ECM of varying density [13]. Such experimental data were used as a motivation to simulate a variety of spheroid behaviors *in silico* (see Fig 1). Using our previously developed platform “Cells in Silico” (CiS) [21], we performed multiple simulations of 3D tumor spheroids, and for this study, we want to highlight a subset of four spheroid phenotypes: “spherical”, “spherical with far gaslikes”, “deformed”, and “disordered”. These phenotypes emerged from different combinations of the aforementioned parameters (see Sections 2.1 and 4.2), and will be used as examples throughout.

In the following, we will first outline our work with CiS towards simulating spheroids and arriving at the four phenotypes. Then, we will describe the spatial features that we extracted from individual simulated and experimental spheroids. Next, we will discuss our strategy for comparing these features between multiple spheroids, including the derivation of an overall deviation score, and how it can be tuned for a specific use case. After validating our strategy via a transformation study, we will show comparisons between exclusively simulated spheroids, exclusively experimental spheroids, and comparisons between simulated and experimental spheroids. We will conclude by evaluating the success of our method, and providing an outlook for its further use.

## 2 Results

### 2.1 Adapting CiS to the simulation of spheroids

CiS is a highly scalable general-purpose framework for tissue simulation at subcellular resolution. It extends a Cellular Potts Model (CPM) with an agent-based layer, and allows the description of various properties and phenomena, such as cell-cell adhesion, cell compressibility and cell motility, cell divison, cell mutation and cell-ECM interactions (see Section 4.1 for more details). In order to apply it to the simulation of spheroids, we first defined the simulation system and parameters to be investigated. Previous studies have identified cell-ECM interactions such as adhesion, degradation and remodeling as strong components in facilitating invasion [22–24]. Hence, we focused on the effects of different ECM alignments, cell-ECM adhesion, ECM degradation, as well as self-propelled cell motility (see Section 4.2 for more details). We performed multiple simulations of 3D tumor spheroids placed in a coarse-grained, rigid ECM. In our model, this ECM can be both adhered to and degraded by cells, but due to its rigidity, the alignment of the fibers remains constant (see Section 4.2 for more detail). Since it is known that tumors remodel their ECM, and ECM alignment is one of the main drivers of invasion [24], we decided to include a radially aligned ECM in our studies (see Fig 1a). Thus, we performed multiple simulations of spheroids at different model parameters (see Table 2). Within this parameter space, we observed four spheroid phenotypes, which were connected by singular differences in parameter values (see Table 1). These were the following:

**Table 1:** Differences between the four simulated phenotypes used throughout this study.

Phenotype	Cell-ECM adhesion	ECM degradation period	Motility magnitude
Spherical	50	∞ (disabled)	0
Deformed	**450**	∞ (disabled)	0
Spherical with far gaslikes	450	**5000 MC steps**	0
Disordered	450	5000 MC steps	**100**

Listed are the three parameters in which the phenotypes differed from each other. The remaining parameters were the same for all phenotypes, and were as follows: ECM density: 1; ECM alignment: radially aligned; Cell-cell adhesion: 50; Random walk persistence: 0; cell division: enabled.

**Table 2:** Simulated parameter space from which the four phenotypes were obtained.

Parameter	Values
ECM density	1, 2
ECM degradation period	1000 MC steps, 5000 MC steps, ∞ (disabled)
ECM alignment	unaligned, radially aligned
Cell-cell adhesion	50, 100
Cell-ECM adhesion	50, 450
Motility magnitude	0, 50, 100
Random walk persistence	0, 0.3, 0.5
Cell division	enabled

Listed are the number of values per parameter, as well as the total number of parameter combinations resulting from this (see section 4.2 for more detail). For each simulation, five replicates were simulated.

#### Spherical

Cell-cell adhesion dominated, and the cells remained in a spherical arrangement, with a relatively smooth surface of the spheroid bulk throughout the simulation.

#### Deformed

Due to a strong increase of the cell-ECM adhesion strength, cells adhered to and moved along the radially aligned fibers. Through a combination of cell division filling gaps and cell-cell adhesion keeping the bulk intact, the spheroid lost its spherical shape and exhibited protrusions along the ECM fibers.

#### Spherical with far gaslikes

Through a combination of high ECM degradation rate and cell-ECM adhesion, cells in the outermost layer of the spheroid moved into the ECM faster than by adhesion alone. This resulted in a halo of singular cells around an approximately spherical spheroid bulk.

#### Disordered

The cells dissociated from each other due to high self-propelled motility. Subsequently, the spheroid integrity was lost.

These four phenotypes visually differed between each other (see Fig 1, and the next step of our investigation was to quantify these differences.

### 2.2 Individual features

We focused on the analysis of spatial properties of tumor spheroids. For this, we utilized data containing the three-dimensional positions of all individual cell centers at one given point in time. For the analysis of these data we considered various features which could be used individually or as an overall deviation score (see Section 2.4). These features and their applicability are highlighted in the following.

#### Cell density distribution

**Fig 2:**
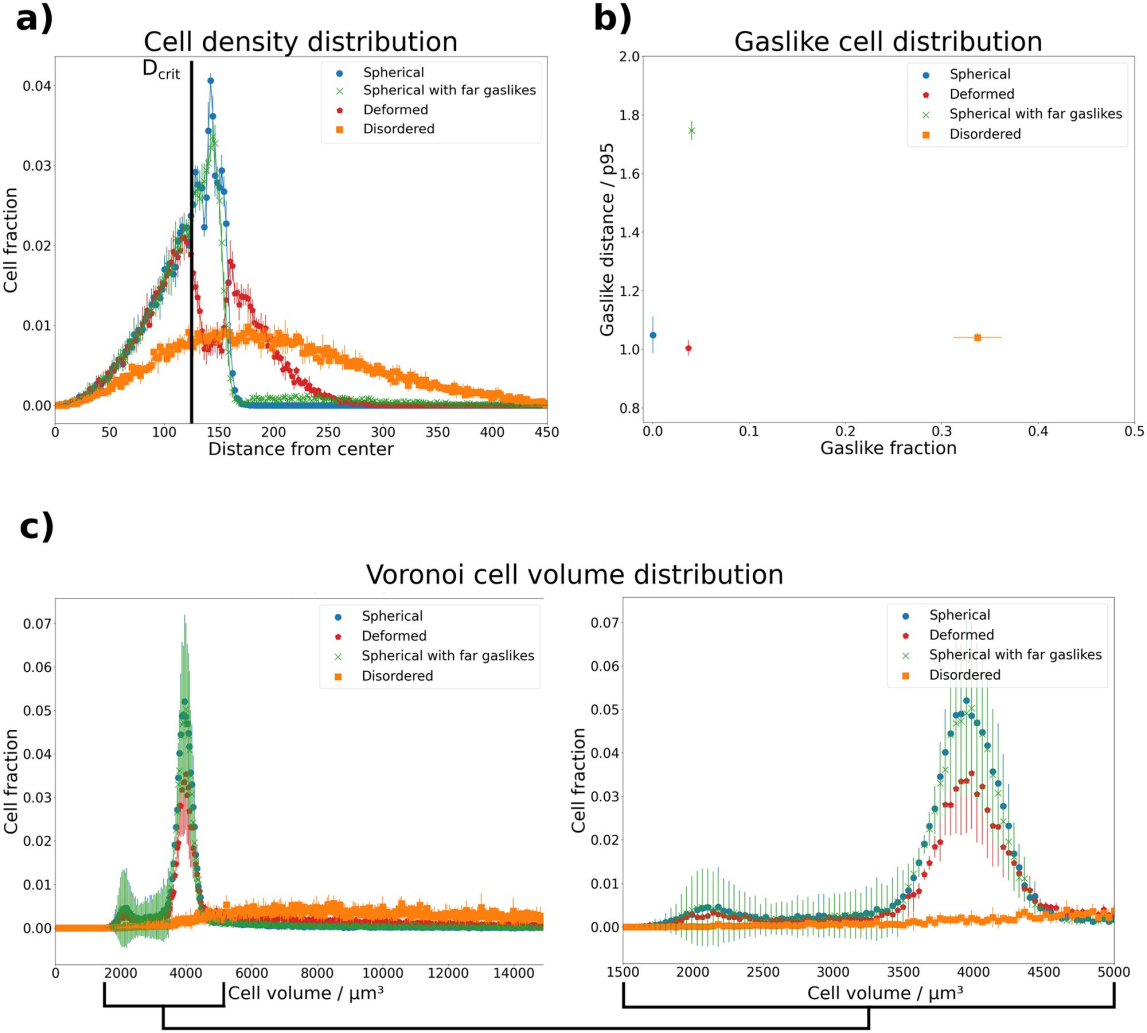
Visualization of cell based features extracted from simulation data for four different phenotypes. **a) Cell density distribution**. Shown is, averaged over all replicates of each phenotype, the fraction of cells within spherical layers around the spheroid center *versus* the radii of these layers. The “spherical” phenotype shows a steep drop at a radius of 150 μm, while the “deformed” and “disordered” phenotypes show a long-tailed distribution. The “spherical with far gaslikes” phenotype behaves similar to the “spherical” phenotype, except for a non-zero density above 175 μm. **b) Gaslike cell distribution**. Shown are the average fractions of gaslike cells according to Eq 1 *versus* their normalized average distance to the spheroid center. The fraction of gaslikes exhibited by the “spherical”, “spherical with far gaslikes” and “deformed” phenotypes is similar, but the distance from the spheroid center is far greater for the “spherical with far gaslikes” phenotype. The “disordered” phenotype on the other hand contains many cells classified as gaslikes across the entire spheroid volume. Their normalized average distance from the center evens out to a value slightly above 1. **c) Voronoi cell volume distribution**. Shown are histograms of the average Voronoi cell volumes found in the four phenotypes. The “spherical” phenotype shows a sharp peak around a volume of 4000 μm^3^, and a smaller peak around a volume of 2000 μm^3^. The “spherical with far gaslikes” and “deformed” phenotypes show a similar behavior, with a slightly more pronounced tail towards larger volumes. Finally, the “disordered” phenotype shows volumes distributed over a wide range. The range between volumes of 1500 μm^3^ and 5000 μm^3^ is magnified on the right to highlight the differences between phenotypes in the two peaks.

Analyzing the distribution of cell density is useful for determining the extent of the bulk of the spheroid, as well as its geometry. For our studies we focused on the so-called central local density, which we defined as the fraction of cells found within spherical layers of constant thickness and increasing radii around the spheroid center. For a uniform spherical distribution of cells, this density is non-zero only within the spheroid bulk (see Fig 2a). Disordered spheroids, on the other hand, exhibit a distribution over a larger domain.

#### Gaslike cell distribution

The detachment of single “gaslike” cells from a spheroid has been a recent focus [13], and can be used to distinguish between ordered and disordered spheroids. However, the assignment strategy of the “gaslike” status needs to be well defined. Kang et al. were able to experimentally measure the spheroid boundary [13], and defined cells outside of this boundary as “gaslike”. Since the spheroid boundary is not tracked in our simulations, we used a definition based on nearest-neighbor distances and distance from the center of the spheroid. The set of gaslike cells *G* as a subset of all cells *C* is thus defined as follows:

$$\begin{array}{c} G=\{c_{i}\in C:d\left(c_{i},O\right)>D_{\text{crit}}\wedge \;\text{min}\;\left(N_{i}\right)>d_{\text{crit}}\}, \end{array}$$ (1)

where *D*_crit_ is the threshold distance from the spheroid center *O*, *N*_*i*_ = {*d*(*c*_*i*_, *c*_*j*_)∣*c*_*j*_ ∈ *C*, *c*_*j*_ ≠ *c*_*i*_} is the set of Euclidean distances between cell *c*_*i*_ and all other cells, and *d*_crit_ is the threshold neighbor distance. The first constraint in Eq 1 provides the context of a bulk structure, and its parameter *D*_crit_ can be selected considering the inflection point of the central local density. The second constraint ensures that only detached cells are defined as gaslikes, and its parameter *d*_crit_ was chosen by considering the mean distance between all cells. Fur our purposes we selected the data by Kang et al. [13] as basis for deriving these parameters: *D*_crit_ = 125 μm, *d*_crit_ = 19 μm. This set *G* can be used to compute various properties, such as the fraction of gaslikes and their average distance from the spheroid center. We combined these two properties in this feature, defining it as a point *p* within the space spanned by them. The first property *p*_x_ describes the fraction of all cells in the spheroid that are detached. The second property *p*_y_ describes the mean distance of the detached cells from the spheroid center.

$$\begin{array}{c} p=(p_{x},p_{y})=(\frac{\left|G\right|}{\left|C\right|},\frac{1}{\left|G\right|}\;\sum_{i}\;\frac{d(c_{i},O)}{p_{95}\left(G^{*}\right)}) \end{array}$$ (2)

where *p*_95_(G*) is the 95th percentile of distances of the non-gaslike cells G* = *C*\*G* from the spheroid center, which serves as a normalization factor. We included only the non-gaslike cells for this normalization factor, because our aim was to define the distance relative to the spheroid bulk. As shown in Fig 2b, the “disordered” and “spherical with far gaslikes” phenotypes can be distinguished from the others using this feature, but it is suited less well for comparing “spherical” and “deformed” spheroids.

#### Voronoi cell volume distribution

The distribution of Voronoi cell volumes within the spheroid serves as a measure of cell deformation, as well as their confinement. To obtain these volumes, we performed a Voronoi tessellation [25] on the cell center point cloud, during which the system was divided into *N*_cells_ regions according to the distances between adjacent cells. It is important to note that the Voronoi cell volumes are not the same as the biological cell volumes, but represent a proxy in which detached cells occupy a significantly larger volume. We generated a histogram of the Voronoi cell volumes, as shown in Fig 2c). Here we observe that the first three phenotypes are distributed sharply around a volume of roughly 4000 μm^3^, with a smaller spike around 2000 μm^3^ and a tail. The volumes of the “disordered” phenotype are evenly distributed over a much wider range. The tail of the first three distributions is a useful artefact of the Voronoi tesselation, as it allows to extract additional information about the bulk spheroid surface.

#### Spheroid surface and surface deformation

**Fig 3:**
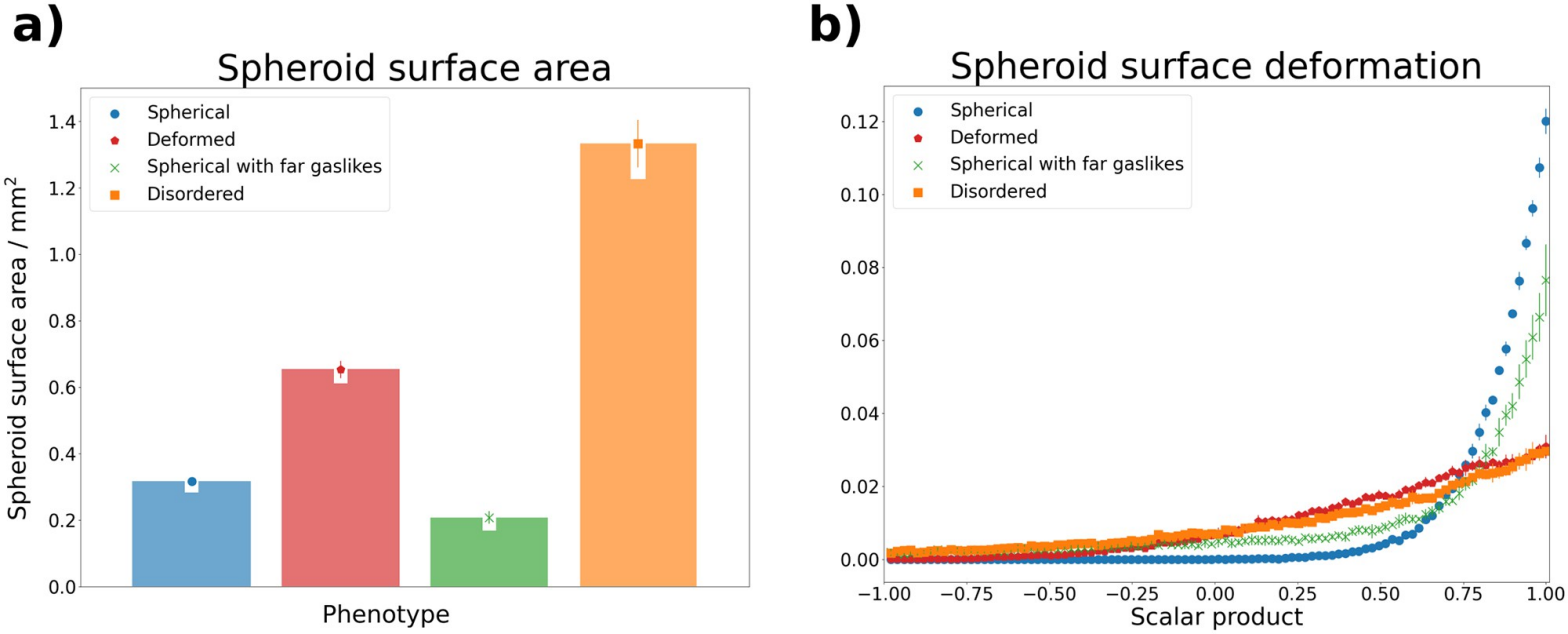
Visualization of spheroid bulk based features extracted from simulation data for four different phenotypes. Surface information was extracted via the marching cubes algorithm [26] (see also section 2.2). **a) Spheroid surface area**. Shown is the average surface area found for each phenotype. The “spherical with far gaslikes” phenotype has the smallest average surface area, due to the spherical bulk containing less cells than that of the “spherical” phenotype. The larger average surfaces of the “deformed” and “disordered” phenotypes are due to their more irregular shape. **b) Spheroid surface deformation**. Shown are histograms of the scalar products between vertex normal vectors and vertex origin vectors, with the origin denoting the center of the spheroid. The vertices were obtained from surface triangulation of the spheroid point cloud and denote points on this surface (see also S4 Fig). The “spherical” phenotype exhibits a sharp peak at scalar products of 1, which is less pronounced for the “spherical with far gaslikes” phenotype. The remaining two phenotypes are spread more widely.

While the cell density distribution provides a measure of the spheroid size, its information about the spheroid shape is limited. To study this in more detail, we needed to approximate the spheroid surface, as we wanted to distinguish deformed spheroid bulk from spherical bulk. We did this via surface triangulation using the marching cubes algorithm [26]. To apply this algorithm we performed some preprocessing of the point cloud data: first, we extracted the set of non-gaslikes G*, as we were only interested in the shape of the spheroid bulk. Next, we obtained a continuous spheroid volume from the remaining points by voxelizing our data. This voxelization was performed by mapping the system onto a 3-dimensional density grid. The density of each grid point was set to 1 if the grid point was closer than a threshold distance to any cell in the point cloud, and was set to 0 otherwise. The resulting region defined by grid points of a density of 1 served as a proxy of the continuous spheroid volume. We then used the marching-cubes algorithm [26] to generate a mesh of triangles which approximated the surface of the continuous volume, a process known as surface triangulation (see also S4 Fig). Finally, we extracted two features from this: first, we calculated the surface area from the triangle mesh (see Fig 3a)), and second, we analyzed the surface deformation by investigating the orientations of the mesh vertices. This was done by calculating, for each vertex, the scalar product between its normal vector and its origin vector, with the spheroid center at the origin (see S4 Fig). Then these scalar products were combined in a histogram. The vertex orientations serve as a measure of deformation, since for a perfect sphere all scalar products are equal to 1, and a deformed sphere results in a more widely spread distribution (see Fig 3b).

By using these features we were able to measure and quantitatively describe different aspects of individual tumor spheroids. This provided a basis on which we could compare two spheroids with each other. Such a comparison required the definition of distance metrics for each feature, which are highlighted in the following.

### 2.3 Individual metrics

To accomodate the different types of output data between features, we required suitable metrics. For the spheroid surface area, which provided a scalar value per spheroid, we used the mean squared error (MSE). For the gaslike cell distribution, which provided a tuple of two coordinates per spheroid, we used the Euclidean distance. Finally, for the distribution-based features, i.e. cell density distribution, Voronoi cell volume distribution and spheroid surface deformation, we used the 1-Wasserstein distance (WSD). The Wasserstein distance is a metric between probability distributions, and is a common sight in mathematics, especially statistics and computer science. The *p*-Wasserstein distance between two probability measures *μ* and *ν* on the metric space $\left(R^{n},d\right)$ is defined as:

$$\begin{array}{c} W_{p}\left(\mu ,\nu \right)≔{\left(\underset{\gamma \in \Gamma (\mu ,\nu )}{\text{inf}}\;\int_{R^{n}\times R^{n}}d{\left(x,y\right)}^{p}\;d\gamma \left(x,y\right)\right)}^{1/p} \end{array}$$ (3)

where Γ denotes the collection of all joint probability measures *γ* with marginals *μ* and *ν* [27], and *d*(*x*, *y*) denotes the metric distance used to the define the metric space. Here, *d*(*x*, *y*) = |*x* − *y*|. An intuitive illustration of the Wasserstein distance can be given by viewing each distribution as a pile of earth of different shape, and considering the amount of work that has to be done to transform one pile into the other. Assuming this work to be equal to the product between the amount of earth that has to be moved and the distance it needs to be moved, the Wasserstein distance is the minimum amount of work that has to be done. Due to this illustration, the WSD is often referred to as “earth mover’s distance” [28].

### 2.4 Combination of multiple metrics

**Fig 4:**
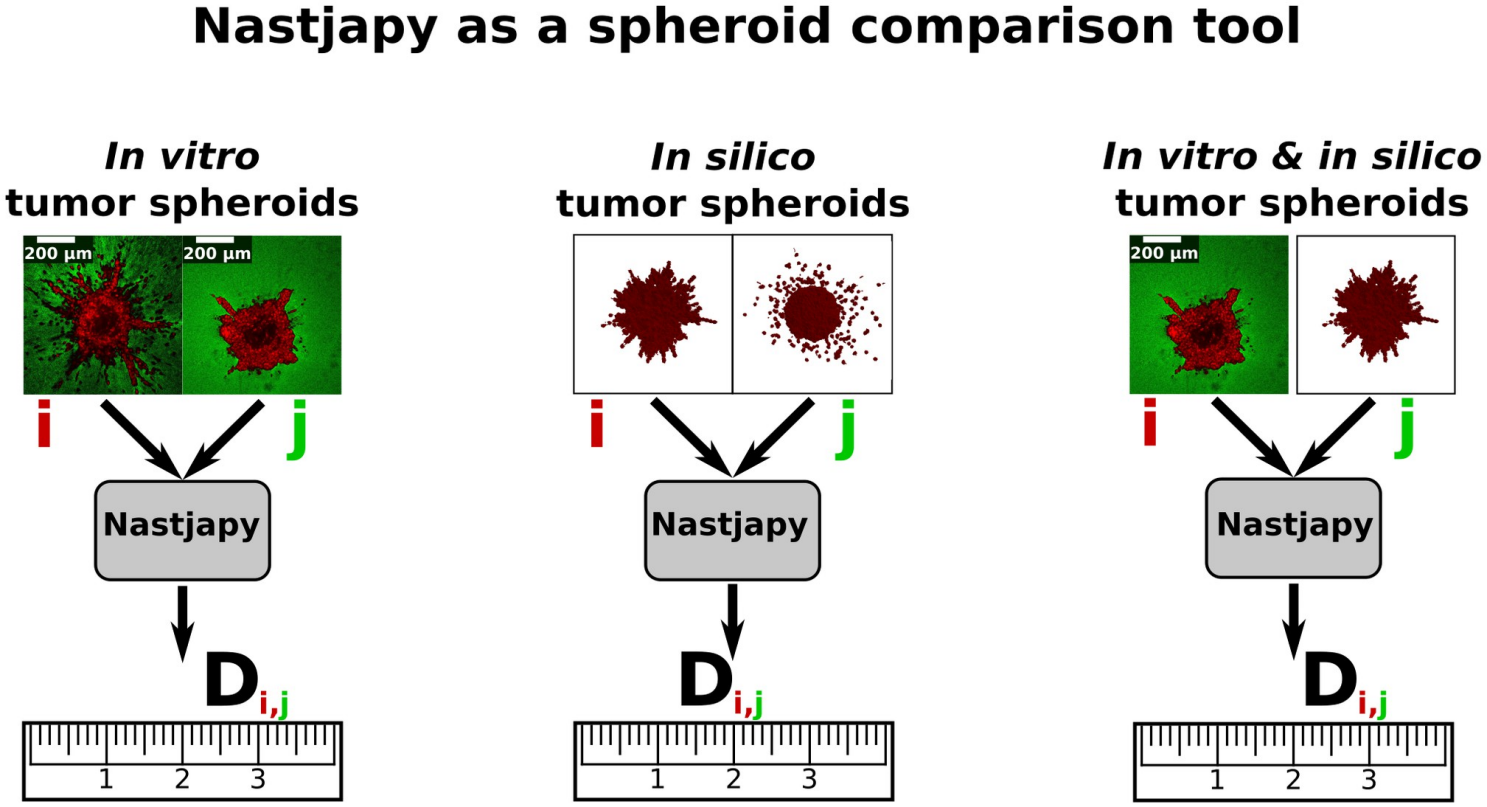
Sketch of the application of *Nastjapy*. An overall deviation score *D*_i,j_ is calculated between two individual spheroids *i* and *j* (see also section 4.4).

At this point, the individual features described in the previous sections could be reliably compared between two spheroids using our defined metrics. Next, one of our main goals was to combine these features and metrics into a single scalar value, which could then serve as an overall deviation score between two spheroids (see Fig 4). This can be seen as a summary statistic, which are also used in Bayesian inference [29]. Many different questions regarding the comparison of tumor spheroids require such a singular scalar. From an experimentalist’s view, this could be the comparison of spheroids cultivated in different conditions, with the goal of quantitatively determining how the change of one experimental variable influences the spheroid growth and invasion pattern. A problem faced by theorists running simulations is how to optimize the model parameters to reproduce experimental results. Both problems require one scalar distance measure like the one we aimed to derive here. Before doing so, we need to address the fact that it is unlikely for such a distance measure to be generally applicable to all types of tumor spheroids and experimental settings. This is due to the high dimensionality of even a single spheroid dataset at a single point in time. To illustrate this, we consider the case of comparing two spheroids, each containing 1000 cells. The desired distance is a function $f:R^{3000}\times R^{3000}\to R$. Such a function will, by design, project many different pairs of spheroids onto the same point in R. This property can hardly be circumvented, and is desired in a distance measure. On the other hand, this also means that the measure has to be carefully selected depending on the use case. Therefore, in addition to defining the overall deviation score here, we will also propose a method to adapt the score to different use cases.

#### Standardization

We first ensured that all metrics were on a similar scale. For this standardization, we used five replicates from each of the four phenotypes from our simulations, and compared each feature, resulting in *N*_spheroids_ = 20 spheroids and *N*_distances_ = 400 metric distances per feature *f*. These distances *d*_i,j,f_ between spheroid *i* and spheroid *j* were then transformed according to Eq 4:

$$\begin{array}{c} d_{i,j,f,\text{std}}=\frac{d_{i,j,f}-\mu_{d,f}}{\sigma_{d,f}} \end{array}$$ (4)

where *μ*_*d*,*f*_ and *σ*_*d*,*f*_ respectively denote the mean and standard deviation across the *N*_distances_ values for each feature *f*. Since this standardization may lead to values of *d*_i,j,f,std_ below zero, and we aimed to define a positive distance for each feature, we further shifted each value by the minimum across all *d*_i,j,f,std_, finally arriving at $d_{i},j,f^{*}$ as defined in Eq 5:

$$\begin{array}{c} d_{i,j,f}^{*}=d_{i,j,f,\text{std}}+\left|\text{min}\left(\left[d_{1,1,f,\text{std}},d_{1,2,f,\text{std}},\;\left(\ldots \right),\;d_{N_{\text{spheroids}},N_{\text{spheroids}},f,\text{std}}\right]\right)\right| \end{array}$$ (5)

#### Overall deviation score and use case adaptation

Next, we defined the overall deviation score *D*_i,j_. This definition entailed merging the previously standardized $d_{i,j,f}^{*}$ via the following linear combination:

$$\begin{array}{c} D_{i,j}\sum_{f=1}^{N_{\text{features}}}\lambda_{f}\cdot d_{i,j,f}^{*}, \end{array}$$ (6)

where λ_f_ denote the weight factors for each feature, i.e. how much it contributes to the final deviation score *D*_i,j_. In order to optimize these values, we once again turned to our simulated phenotypes and their five respective replicates. Because we wanted to ensure the deviation score to distinguish between different phenotypes, we required values of λ_f_ that minimized the intra-phenotype deviations and maximized the inter-phenotype deviations. This can be formulated as a maximization problem:

$$\begin{array}{c} \underset{\left\{\lambda_{f}\right\}}{\text{max}}\sum_{f=1}^{N_{\text{features}}}\lambda_{f}\sum_{k=1}^{N_{\text{phenotypes}}}(\underset{\text{inter-phenotype}}{\underset{︸}{\sum_{i\in P_{k}}\sum_{j\notin P_{k}}d_{i,j,f}^{*}}}-\underset{\text{intra-phenotype}}{\underset{︸}{\sum_{i\in P_{k}}\sum_{j\in P_{k}}d_{i,j,f}^{*}}}), \end{array}$$ (7)

where *P*_*k*_ is the set of all spheroids of phenotype *k*. This optimization procedure can be interpreted as an inverse clustering. During clustering, the property described in Eq 7 is maximized by assigning individuals to a cluster. In contrast to this, our method uses prior clustering information to optimize the metric space itself. This shows resemblance to methods of contrastive learning, with the notable difference that we use a linear model in our approach [30]. We argue, that adjusting the weighting of features according to their relevance to the formation of predefined clusters will allow to more strongly distinguish between those clusters. Assuming that phenotypes are correctly grouped, maximizing Eq 7 already ensures that each λ_*f*_ > 0. Additionally, we decided on the following constraint:

$$\begin{array}{c} \sum_{f=1}^{N_{\text{features}}}\lambda_{f}^{2}=1 \end{array}$$ (8)

This constraint is important to prevent the optimization procedure from collapsing towards the trivial solution of setting λ_*f*_ → ∞. It also fixes each λ_*f*_ to the domain [0, 1]. Furthermore, using the square of each λ_f_ minimizes the influence of outliers across features. The optimization of Eq 7 under consideration of this constraint was performed using the Sequential Least Squares Programming (SLSQP) method implemented in the SciPy package [31] The contribution of each feature to the overall deviation score resulting from the optimization of the λ_f_ is shown in Table 3.

**Table 3:** Fitted weight factors for each feature contributing to the overall deviation score between two spheroids.

Feature	Cell density distribution	Gaslike cell distribution	Voronoi cell volume distribution	Spheroid surface area	Spheroid surface deformation
**λ_f_**	0.41	0.50	0.43	0.34	0.52

The values were obtained by maximizing Eq 7, with the simulated phenotypes serving as a calibration set (see Section 2.1).

### 2.5 Validation: Transformation study

Validating a metric, such as the one derived in this work, requires a set of data examples with known relation. For this purpose, we designed a transformation study, in which we generated multiple point clouds from a reference spheroid using transformation functions. These functions were selected in such a way, that for a useful metric we expected higher distances between reference and transformed point cloud for higher transformation strengths. On the other hand, the metric has to remain invariant under transformations related to the frame of reference, e.g. rotation or translation of the point cloud. Therefore, we also included these transformations. Hence, we tested for the following properties:

1. invariance under rotation and translation,
2. monotony within the domain of interest: a small deviation from an original spheroid shall result in a lower distance than a large deviation.

We investigated these properties for the five described features and the overall deviation score by appling four transformations to a spheroid $S=\left\{\vec{P}\right\}|\vec{P}\in R^{3}$ of the “spherical” phenotype. The transformations were represented by functions in the space of point clouds T:S×R→S. With this approach, we aimed to verify both of the above properties. Invariance is shown, when the distance does not depend on the strength of the transformation. Similarly, monotony is shown, when the distance metric grows monotonously with the transformation strength. The four transformations that we used were the following:

#### Rotation

Rotating each cell of the spheroid by a given angle *α* around an arbitrary axis *i* through the spheroid center:

$$\begin{array}{c} T_{R}\left(S,\alpha \right)=\left\{R_{i}\left(\alpha \right)\vec{P}|\vec{P}\in S\right\}, \end{array}$$ (9)

where *R*_*i*_(*α*) is the rotational matrix.

#### Noise

Adding a random vector drawn from a standard uniform distribution to the position of each cell:

$$\begin{array}{c} T_{N}\left(S,\alpha \right)=\left\{\vec{P}+\alpha \cdot \vec{X}|\vec{P}\in S,\vec{X}∼U_{3}\left(0,1\right)\right\}, \end{array}$$ (10)

#### Deformation

Translating each cell along the radial vector of the spheroid, modulated by the spherical angles of the cell’s position:

$$\begin{array}{c} T_{D,\omega }\left(S,\alpha \right)=\left\{\vec{P}+\alpha \cdot {\hat{e}}_{r}\cdot (\text{cos}\;\omega \varphi +\text{sin}\;\omega \theta )|\vec{P}\in S\right\} \end{array}$$ (11)

This deformation can be interpreted as adding ripples with frequency *ω* and amplitude *α* to the spheroid surface.

#### Scaling

Multiplying the position of each cell by its distance to the center of the spheroid:

$$\begin{array}{c} T_{S}\left(S,\alpha \right)=\{\alpha \cdot \|\vec{P}\|\cdot \vec{P}|\vec{P}\in S\} \end{array}$$ (12)

This transformation affects cells with a larger distance to the spheroid center more strongly than those close to it. The spheroid density is therefore not conserved.

**Fig 5:**
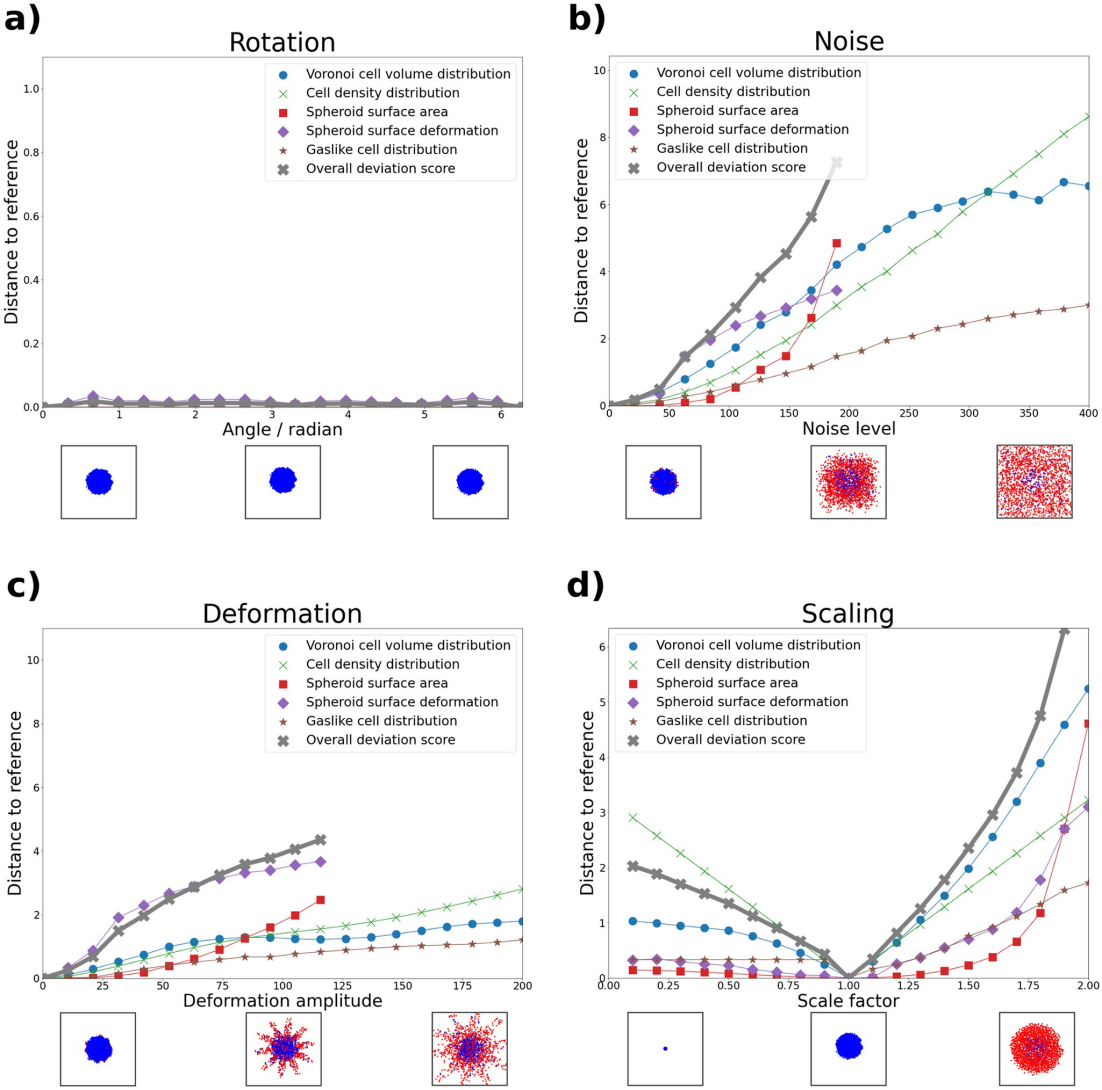
Feature comparison for spheroid point clouds resulting from four different transformation functions. Shown are the standardized metric distances between the un-transformed reference spheroid and an increasingly transformed version for each data feature. In addition, the combined deviation score is depicted in gray crosses for each transformation (see Section 2.4). Below each subfigure, we provide a top-down view snapshot of the spheroid at three levels of transformation. Blue cells are classified as non-gaslike, and red cells are classified as gaslike. **a) Rotation**. Except for negligible changes in the spheroid surface deformation feature, we observe no change at increasing rotation angle. This supports rotational invariance of our features. **b) Noise**. For each feature, the distance increases at increasing noise level. Due to the loss of a solid core at high noise levels, the spheroid surface area and deformation features are no longer sensible, and were therefore cut. The deviation score increases approximately linearly up to a noise level of 200, at which point the features related to the spheroid surface area were cut. **c) Deformation**. Similar behavior to b) is observed here. Above a deformation amplitude of 120 the spheroid point cloud still contains cells classified as non-gaslike but loses its solid core. Surface area and deformation values were therefore cut above this threshold. The deviation score increases approximately linearly up to a deformation amplitude of 120. **d) Scaling**. We observe increased distances both for scale factors below and above 1. Due to the fixed values of *D*_crit_ and *d*_crit_ (see Eq 1), the gaslike distribution feature is scale-dependent, and also varies here. For scale factors below 1.0, no gaslikes were found, and therefore the values of this feature remained constant. The deviation score increases approximately linearly both for scale factors smaller and larger than 1.

A visual example for each of these four transformations is provided in Fig 5. For completeness, we also mention the translation transformation, which is defined as follows:

#### Transformation

Changing the position of each cell by the same vector $\vec{\alpha }$

$$\begin{array}{c} T_{T}\left(S,\vec{\alpha }\right)=\left\{\vec{P}+\vec{\alpha }|\vec{P}\in S\right\} \end{array}$$ (13)

We do not show it however, because translational invariance is ensured. This is because all features depend only on relative and not absolute distances, and this transformation conserves relative distance. Rotational invariance was expected due to the rotational symmetry of the underlying features. Nonetheless, we wanted to test whether artifacts, produced by the voxelization for features related to the spheroid surface (see Section 2.2), had any notable effect. For this reason, we included the rotation transformation. The remaining transformations were chosen to validate the monotony of the deviation score.

We applied each transformation at increasing strength and compared the resulting spheroid with the untransformed version. The results of this are shown in Fig 5. Starting with the rotation transformation in subfigure a), we observed no change in the feature distances at increasing rotation angle, except for negligible changes in the spheroid surface derformation. This underlines the rotational invariance of our features. For the other three transformations, we observed monotony in all cases. Those features related to the spheroid surface could not be meaningfully extracted when the spheroid bulk was disrupted, i.e. at high degrees of the noise and deformation transformations (subfigures b) and c)). The gaslike distribution feature distance remained constant for small values of the scaling transformation (subfigure d)), since no cells were classified as gaslike here. Aside from these edge-cases, our features behaved robustly. It is interesting to note that the overall deviation score scaled approximately linearly with the transformation strength within the domain of interest, excluding the aforementioned extreme cases. This property can be viewed as a stronger version of the monotony property. Importantly, this was not used as a constraint when optimizing the weights, but emerged from the procedure itself.

### 2.6 Validation: Comparing simulated spheroid phenotypes

**Fig 6:**
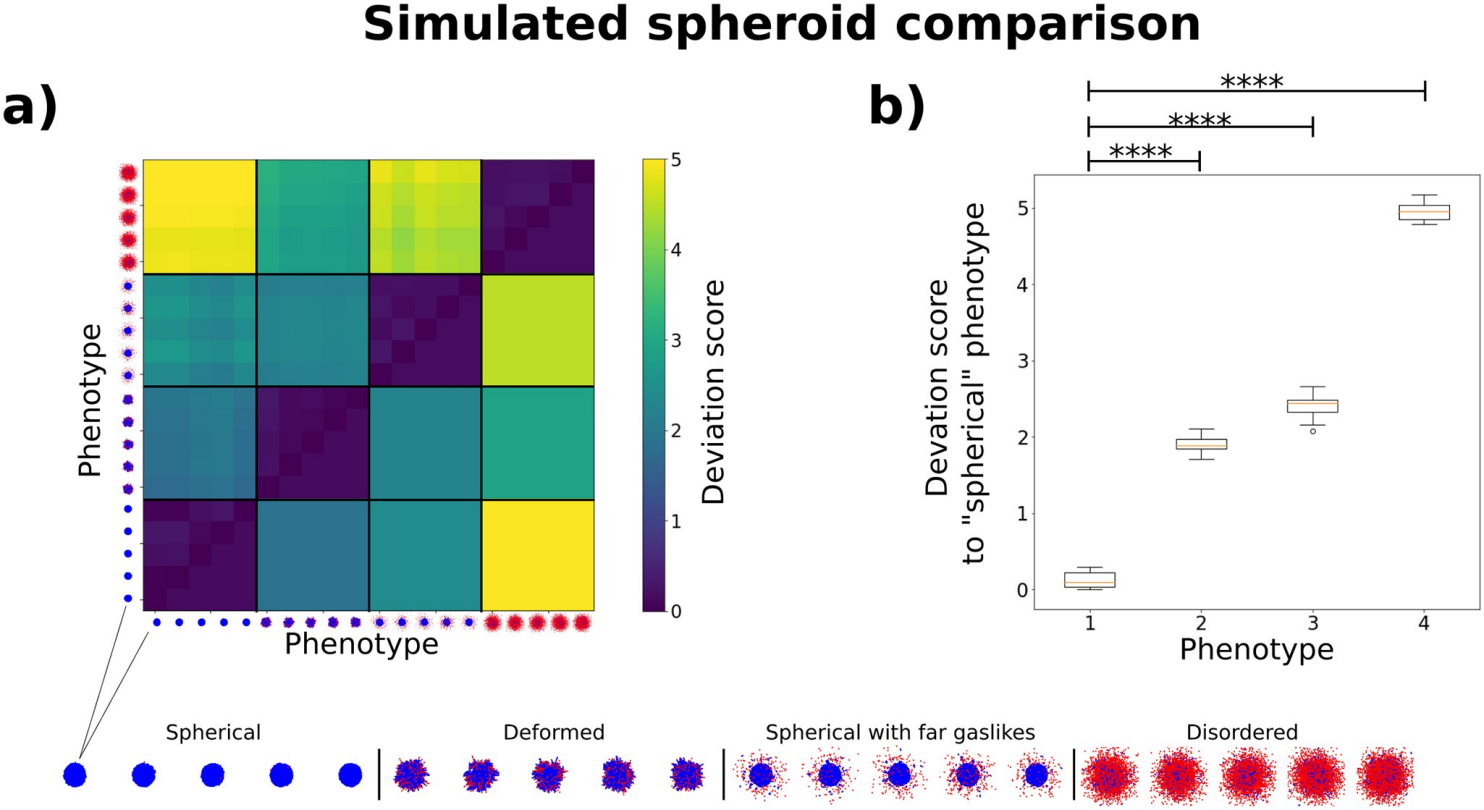
Deviation score comparison for four simulated spheroid phenotypes. **a)** Shown are the deviation scores for five replicates of each phenotype on the upper triangle, and the average deviation score over all replicates of each phenotype on the lower triangle. A top-down view of the spheroid point cloud for each replicate is shown next to the respective row/column. Blue cells are classified as non-gaslike, and red cells are classified as gaslike. For better comparison, an enlarged version of each spheroid was placed at the bottom of the figure. We observe the highest deviation between the “disordered” phenotype and the others, with the maximum deviation between the “spherical” and the “disordered” phenotypes. The “spherical”, “spherical with far gaslikes” and “deformed” phenotypes, which are more similar from a visual perspective, show a smaller deviation score using our analysis, but are nonetheless distinguishable. **b)** Box plots of the deviation score values between the “spherical” phenotype and each other phenotype. The values used here correspond to those used for the lowest row of subfigure a). We observe that the deviation scores for the “spherical” phenotype compared with the other phenotypes consistently lie above the maximum deviation score of the “spherical” phenotype compared with itself. Significance was determined using Welch’s t-test.

As a second way to validate our methods, we now moved to the comparison of simulated spheroids. We chose the final simulation state, after 250 000 MC steps, of five new replicates from each phenotype. Importantly, these were not the same replicates which we used earlier for the calibration of the weight factors. We calculated the overall deviation score for each pair. As shown in Fig 6a), we compared individual replicates (upper triangle), and we also combined replicate comparisons into an average phenotype deviation score (lower triangle). Importantly, while it is questionable whether the “disordered” phenotype is a biologically occuring configuration (see section 3), we chose to include it to serve as a phenotype maximally distant to the others. The deviation score was lowest when a phenotype was compared with itself, and highest, when any phenotype was compared with the “disordered” one. The “spherical”, “deformed” and “spherical with far gaslikes” phenotypes showed a smaller deviation score between each other, but were nonetheless distinguishable. This is underlined in subfigure b), in which we show box plots of the overall deviation scores between the “spherical” phenotype and the others. Here, each phenotype comparison was clearly distinct from the comparison of the “spherical” phenotype with itself (see also S1 Fig). Significance was determined using Welch’s t-test.

### 2.7 Validation: Comparing experimental spheroids

**Fig 7:**
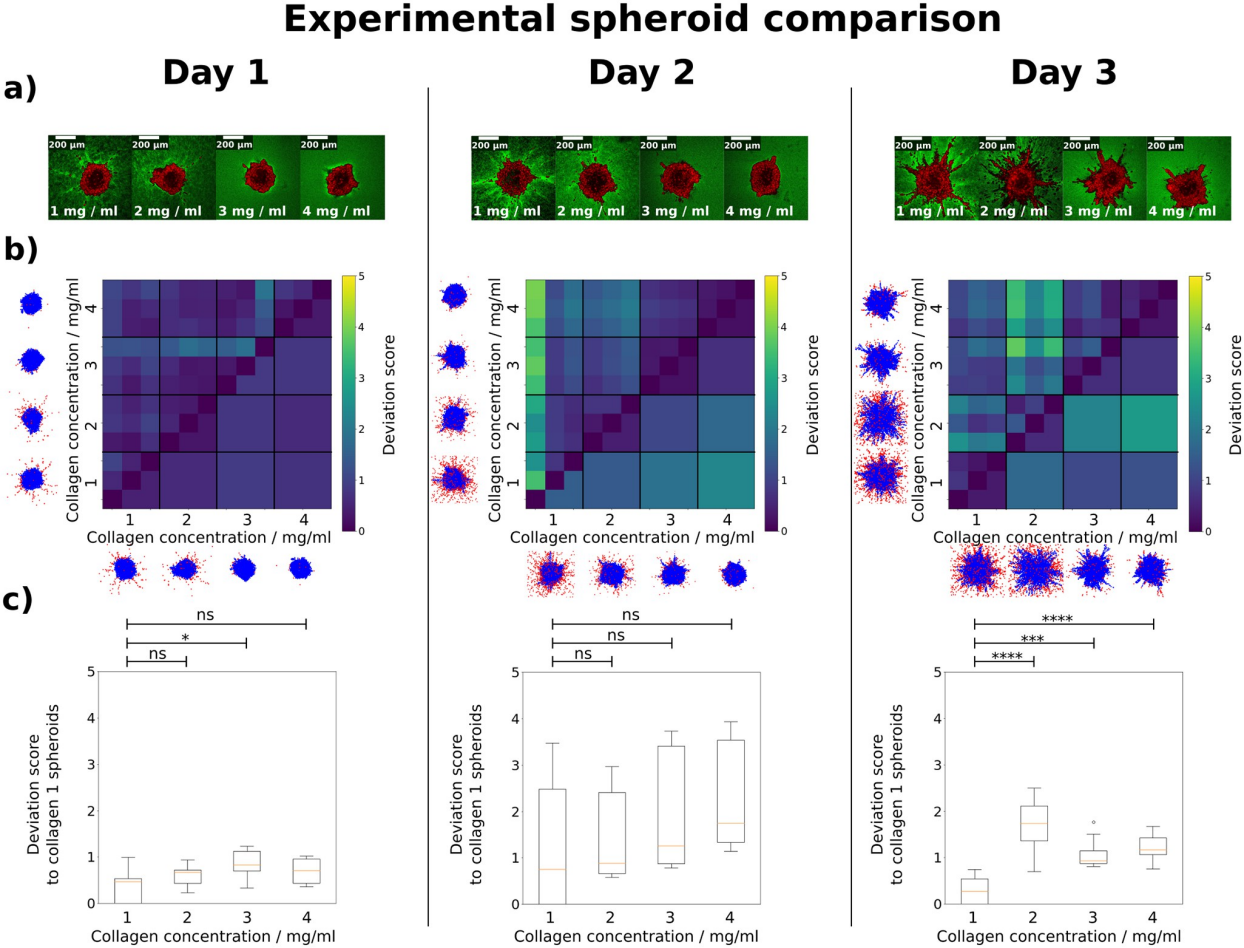
Deviation score comparison for *in vitro* MDA-MB-231 spheroids cultured in four collagen concentrations *c* (data provided by Kang et al [13]). **a)** 2D cross-sections of 3D multiphoton microscopy image stacks depicting one replicate of each collagen concentration [13]. Spheroids were imaged at one, two or three days after embedding in collagen, and were then fixed and imaged (see Section 4.3). **b)** Deviation score comparison between all spheroid samples. For each day, the deviation scores for three replicates of each collagen concentration are shown on the upper triangle, and the average deviation score for each collagen concentration is shown on the lower triangle. A top-down view of the spheroid point cloud for a representative replicate of each collagen concentration is shown next to each heatmap (see S2 Fig for all replicates). Blue cells are classified as non-gaslike, and red cells are classified as gaslike. Due to matching initial conditions, we observe low deviation scores between spheroids grown for one day. These differences increase at day 2, where we observe an approximately linear increase of the average deviation score from *c* = 1 mg/ml to *c* = 4 mg/ml. Finally, at day 3, we observe the lowest deviation between *c* = 3 mg/ml and *c* = 4 mg/ml. This underlines the findings by Kang et al., who observed a transition in invasion behavior between 2 mg/ml and 3 mg/ml. **c)** Deviation score box plots from spheroids grown for one, two and three days respectively. The box plots for each day show the deviation score values between *c* = 1 mg/ml and each other concentration. These values correspond to those used for the lowest rows in b). We observe that for spheroids grown for one and two days, the deviation score values of *c* = 1 mg/ml compared with itself are similar to the deviation score values of *c* = 1 mg/ml compared with the other concentrations. The differences are nonsignificant and therefore not sufficient to clearly distinguish between the concentrations. This changes at day three, where each concentration shows a higher deviation score to *c* = 1 mg/ml than *c* = 1 mg/ml compared with itself. Significance was determined using Welch’s t-test.

To demonstrate the fact that our deviation score can also be used for experimental data, we again turned to the dataset on which we based our initial simulations. Kang et al. previously investigated the invasive behavior of tumor spheroids cultured in increasing collagen concentrations [13]. Their data set contained cell-resolved 3D snapshots of spheroids in four different collagen concentrations (1–2-3-4 mg/ml) at different times (days 1–2-3) during invasion (see Fig 7a)). For each collagen concentration and day of culture, data from three individual spheroids were acquired using a combination of optical clearing and multiphoton microscopy. Since the optical clearing procedure requires fixation, data from successive days of culture share the same initial conditions but do not originate from the same spheroid. For more details, see Section 4.3.

In order to keep the scale consistent throughout this study, we used the same standardization and weight factors as for our simulated spheroids. We compared the spheroids grown for one day, two days, and three days, and visualized them in Fig 7. Similar to Fig 6a), in Fig 7b) we show, for each growth duration, the deviation scores for individual replicates on the upper triangle, and the average deviation score over a set of replicates on the lower triangle. For illustrative purposes and consistency, we have kept the color bar range the same as before. In subfigure c), we show box plots of the deviation score values between collagen 1 and each other concentration. As expected, we observe low to nonsignificant devation between the day 1 spheroids, both in subfigure b) and c). This changes at day 2, where the difference between collagen concentrations becomes clearer. However, while the difference is visible in the average deviation score in subfigure b), there is still strong overlap between the values across concentrations. Furthermore, in subfigure c) we see that the differences are not significant. Finally, on day 3, we see the highest difference between the collagen concentration of 2 mg/ml versus the other concentrations, while spheroids of collagen concentrations 3 and 4 mg/ml are most similar to each other. This is consistent with qualitative observations from subfigure a), and from prior quantifications by Kang et al. (cf., Fig 4 c) in [13]) who observed a sudden transition in single cell individualization during invasion between collagen concentrations of 2 and 3 mg/ml. Importantly, as seen in subfigure c), the deviation score reliably distinguishes between the concentrations at day 3.

### 2.8 Comparing simulated and experimental spheroids

**Fig 8:**
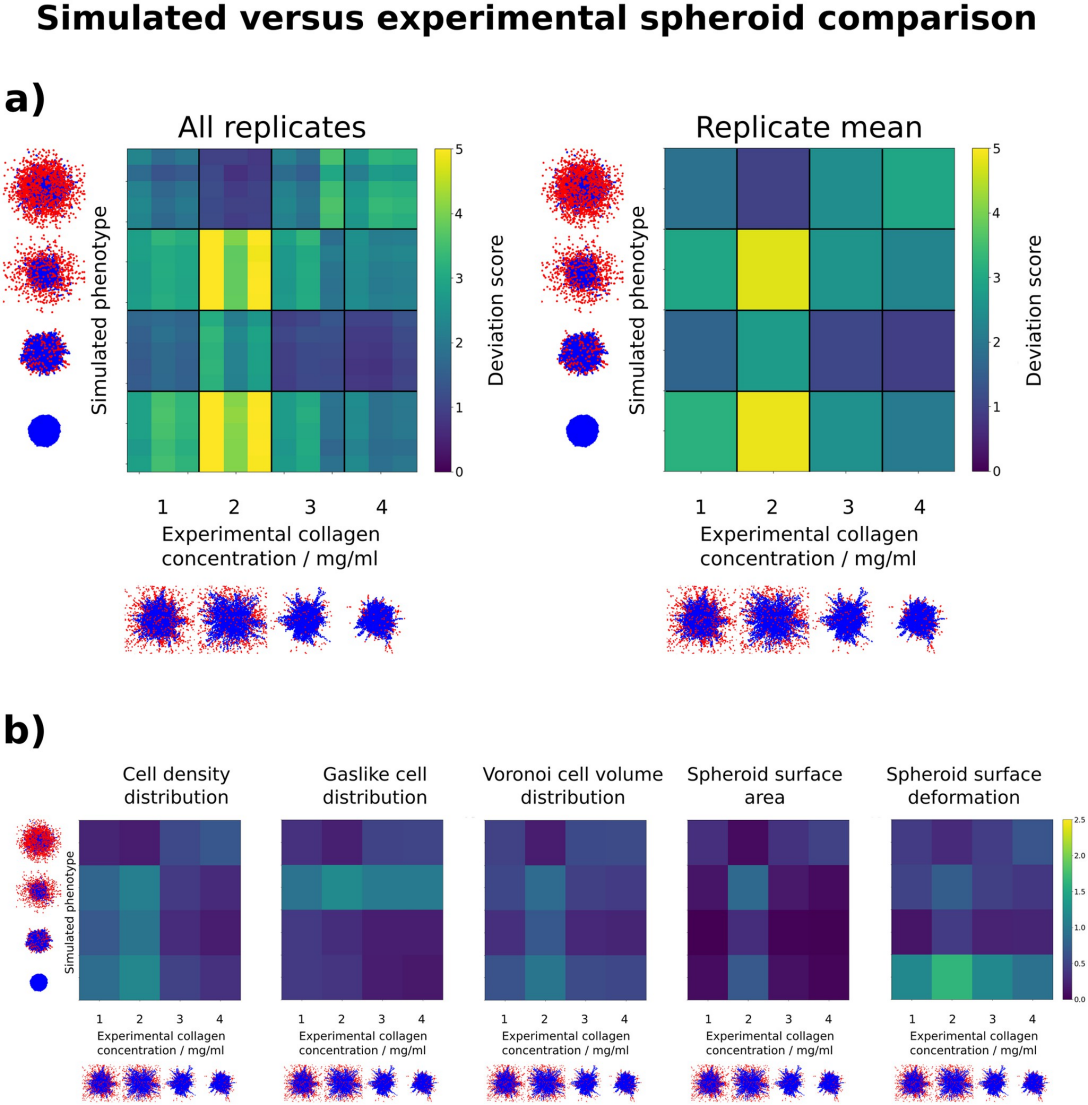
Deviation score comparison between *in vitro* spheroids grown in media at four different collagen concentrations *c* (data provided by Kang et al [13]), and *in silico* spheroids exhibiting four different phenotypes, simulated by us. **a)** Shown are the deviation scores between three replicates of each collagen concentration, grown for three days, and five replicates of each simulated phenotype, simulated for 250 000 MC steps. A single MC step corresponds to roughly 1 s of real time in this context. Each individual deviation score is shown on the left, and the average within a pairing of collagen concentration and phenotype is shown on the right. A top-down view of the spheroid point cloud of a representative replicate for each collagen concentration / phenotype is shown next to both heatmaps. Blue cells are classified as non-gaslike, and red cells are classified as gaslike. See S2 Fig for an enlarged view of all experimental spheroid point clouds, and Fig 6 for an enlarged view of all simulated spheroid point clouds. We observe the highest average deviation scores between *c* = 2 mg/ml and the “spherical” phenotype. The lowest average deviation score is found between *c* = 4 mg/ml and the “deformed” phenotype **b)** Individual metric distances for each of the features constituting the overall deviation score. Shown are the standardized and weighted metric distances between three replicates of each collagen concentration, grown for three days, and five replicates of each simulated phenotype, simulated for 250 000 MC steps. A single MC step corresponds to roughly 1 s of real time in this context. Due to the overall deviation score being a sum of all weighted feature distances, the color range has been adjusted here. The highest deviation score observed in a) is a combination of high metric distances in all features, especially the spheroid surface deformation. On the other hand, the lowest deviation score observed in a) stems from overall low values, especially in the spheroid surface area.

Finally, to show another aspect for which the deviation score may be used, we applied our analysis method to the comparison between simulated and experimental spheroids. For this, we used both our simulated phenotypes, and the experimental data from spheroids grown for three days (see Section 2.7). Since the simulation parameters used here were not fitted to the data, but represented default parameter sets, we did not expect a high degree of similarity. On the other hand, this provided an opportunity to investigate both the overall deviation score and the underlying feature distances, and to demonstrate how the differences in spheroid morphology manifested themselves within the features. In subfigure a) of Fig 8, we show the comparison between each experimental replicate (horizontal) and each simulated replicate (vertical) on the left side. On the right side we show the average within replicates. Here, we observed the highest deviation scores between collagen density 2 and both “spherical” (S) and “spherical with far gaslikes” (SFG) spheroids. Visually, this is sensible when comparing the 2D images of the replicates (see also S2 Fig for this); the round shapes of the S and SFG spheroids differ strongly from that of spheroids in 2 mg/ml collagen, as does the number and location of cells classified as gaslike. Furthermore, we observed the lowest deviation scores between spheroids in 4 mg/ml collagen and “deformed” spheroids. Here, the spheroid shape visually matched much better between the replicate images. The deviation score between spheroids in 2 mg/ml collagen and the “disordered” (D) phenotype is also low. These visual differences and similarities are reflected in our features, as seen within subfigure b). Here we decomposed the overall deviation score back into its components, and thereby show the influence of each feature on it. To improve visibility, the color bar range maximum is set to half of that shown in subfigure a). We see that the distance between the collagen density 2 spheroids and the SFG phenotype is noticable for all features. Of these, the gaslike cell distribution and the spheroid surface area exhibit especially high distances. Conversely, the high deviation score of the spheroids with collagen concentration 2 mg/ml to the S phenotype stems mostly from the difference in surface deformation, and is less pronounced in the other features. Regarding the lowest observed deviation scores, collagen density 4 spheroids and “deformed” spheroids match comparatively well for each feature, and the spheroid surface area distance between them is the lowest of all. Collagen concentration 2 mg/ml and “disordered” spheroids also exhibit a low deviation score between each other, due to low difference in cell density distribution, Voronoi cell volume distribution and spheroid surface area.

### 2.9 Nastjapy

During our derivation of the overall deviation score and its application to various data, we developed the Python package *Nastjapy*. Through this, we wanted to facilitate the use of our procedure by others. The package can be found at http://www.gitlab.com/nastja/nastjapy. *Nastjapy* allows the investigation of spheroids and other single-cell resolved data from different origins. It thereby unifies the analysis pipeline for simulated data and data from multiple experimental sources. See Section 4.4 for more details.

## 3 Discussion and conclusions

Both experimentalists and theorists produce data concerning tumor spheroids. However, both the quantitative comparison between different experiments or simulations, and processes such as fitting simulations to experimental data, are hindered by the lack of an adaptable distance measure that captures the similarity of the spatial features of two spheroids. We aimed to solve this issue via the following steps. First, we proposed a set of five relevant spatial features, which could be extracted from spheroid point clouds. Next, we devised metrics to compare each feature, and combined all metrics into an overall deviation score. We also provided an optimization scheme which could be used to adapt the deviation score to the specific use case. For this, we turned to four *in silico* spheroid phenotypes which emerged from our simulations, and used them to standardize and combine the metrics into the overall deviation score *D*_*i*,*j*_. We did this by weighing individual metrics differently while maximizing a phenotype separation property (see Eq 7). We characterized the behavior of our features by applying four different transformations to a point cloud obtained from a spherical simulated spheroid. We were able to confirm rotational invariance by analyzing the “rotation” transformation, and monotony for the others. While the features related to the spheroid surface showed some instabilities for higher transformation strengths in the “noise” and “deformation” transformations, this only occured when the point cloud was so disordered that a solid core could no longer be defined. Overall, the behavior of the features was therefore considerered suitable to quantitatively compare the structure of spheroids. Interestingly, the overall deviation score did not only scale monotonously with the strength of the studied transformations, but did so approximately linearly within the domains of interest. This is a useful property for a distance measure, which was not used as a constraint, but instead emerged as a result of our optimization scheme.

During the investigation of our simulated spheroid phenotypes, we found that our deviation score distinguished well between dissimilar spheroid phenotypes, as all phenotypes showed the highest deviation score towards the “disordered” phenotype (see Fig 6). This large distance was a value that was desired, as this phenotype did not have a solid core and was therefore deemed to be least similar to all other phenotypes. The slightly lower deviation score found between the “disordered” and “deformed” phenotypes can be attributed to the fact that our spheroid surface extraction method constructs surfaces for each cluster of cells that is close enough together to be considered solidlike. These surfaces sum up to a large overall surface area, and are highly deformed due to their irregular structure. These features are therefore less meaningful if a singular bulk structure is not present. On the other hand, even glioblastoma spheroids, which originate from one of the most invasive types of cancer, have been shown to retain a bulk structure [32]. We therefore view the “disordered” phenotype as an edge case, but wanted to show it for completeness. Since we observed significant deviation between the other three phenotypes (see Fig 6b) and S1 Fig), we were able to confirm that our strategy is also applicable to more similar spheroids. We therefore consider *D*_*i*,*j*_ to represent a useful metric for the systematic quantification of spheroid similarity. This was further confirmed by our analysis of experimentally measured spheroids generated by Kang et al. [13].

During our analysis of the experimental spheroids, we observed that statistically significant difference between the four collagen concentrations only occured after three days of growth (see Fig 7c). This suggests that there exists a minimum time which is required until differences in the spheroid structure manifest themselves sufficiently to be detected by Nastjapy. On the other hand, this could also be used as a tool to study such timeframes in more detail, e.g. when investigating the response time of drug or radiation treatments.

One of the possible applications of the deviation score is to use it as a reliable objective function for fitting simulated spheroids to experimental data. Therefore, we included a comparison between simulations with unfitted, default parameters, and the aforementioned experimental spheroid data. Here, we were able to highlight, which features contributed most towards each deviation score, and to show that the quantities matched well with a visual comparison. Since the “disordered” phenotype is not present in the experimental data, we expected to see high deviation scores in the top rows of Fig 8a). However, this was not the case, which once again stems from how the surface extraction method functions when a solid spheroid bulk is not present. While we still consider the “disordered” phenotype as an edge-case for spheroids, this represents an opportunity to further develop these features in the future. Distinguishing between singular and multiple bulk structures could become relevant once we apply *Nastjapy* to the analysis of more complex tissues, e.g. organoids.

The features we have defined here admittedly have some limitations. They cannot, for example, measure the dynamics of cell movement over time. Also, spheroids or other tissues we might want to apply this method to, may be composed of multiple different types of cells, and we currently do not distinguish between these. However, since we implemented our strategy in our freely available *Nastjapy* framework (see Section 4.4), it can easily be extended. We aim to further develop this in the future, via incorporating more features. Points of interest would be generating features spanning multiple timesteps, e.g. cell velocity correlation and autocorrelation. Furthermore, we envision features such as the distribution of different cell types, which will enable the application of the analysis scheme to the aforementioned non-spheroid tissue models.

## 4 Materials and methods

### 4.1 Model description

*Cells in Silico* is a framework for simulating the dynamics of cells and tissues at subcellular resolution, which was previously developed by our group [21]. It combines a Cellular Potts Model (CPM) at the microscale with nutrient and signal exchange at the mesoscale and an agent-based layer at the macroscale. This enables detailed capture of individual cell dynamics. Furthermore, as an extension of the *NAStJA* framework [33] its efficiency scales excellently with increasing system size and CPU core number. Hence, CiS has already been used for simulating tissues composing millions of cells [21]. Here, we briefly outline the main properties of the microscale, mesoscale and macroscale layers, and a more detailed description can be found in [21].

#### Microscale

The CPM was developed by Graner and Glazier in 1992 [34], as an extension of the Potts model. In it, a system of lattice points on a regular grid is propagated according to its overall energy. Cells are defined as aggregates of points of the same type (see S5 Fig), and the overall energy of the system is built of multiple components *E*_*i*_, which dictate the morphology of and interaction between the cells. Weighted by coupling factors λ_*i*_, they are combined into the following Hamiltonian:

$$\begin{array}{ccc} H_{\text{CPM}} & =\sum_{i}\lambda_{i}E_{i}\\ & =\lambda_{V}\sum_{c\in C}{\left(v(c)-V(\tau (c))\right)}^{2}\; & \text{Cell}\;\text{volumes}\\ & +\lambda_{S}\sum_{c\in C}{\left(s(c)-S(\tau (c))\right)}^{2}\; & \text{Cell}\;\text{surfaces}\\ & +\sum_{i\in \omega }\sum_{j\in N(i)}A_{\tau \left(c_{i}\right),\tau \left(c_{j}\right)}(1-\delta (c_{i},c_{j}))\; & \text{Cell-cell}\;\text{adhesion} \end{array}$$ (14)

where *c* is a cell from the set of all cells *C*, *τ*(*c*) is the type of cell *c*, *s*(*c*) and *v*(*c*) are the current surface and volume of cell *c*, *S*(*τ*) and *V*(*τ*) are the target surface and volumes of cells of type *τ*, *A* is the adhesion coefficient matrix for all cell types, *N*(*i*) are all lattice points neighboring point *i*, and *δ* is the Kronecker delta. Eq 14 can be extended to include further effects, such as cell motility [35] (see also Section 4.2.

#### Mesoscale

CiS includes the capability of introducing signals or nutrients to the system. These can be exchanged between cells via the cell-cell interface. As this functionality is outside of the scope of this study, we only briefly mention it here and refer the reader to [21].

#### Macroscale

While using the CPM layer allows for excellent reproduction of cell shape and deformation, there are other important cellular functions which are not intrinsically captured. For example, the CPM Hamiltonian does not in itself include the effect of cell division. Furthermore, while self-propelled cell motility can be added to Eq 14 [35], the direction of the motility vector must be periodically updated for each cell, to ensure realistic movement, e.g. via random walk (see also Section 4.2). This requires information on the cell center location, which must be extracted from the CPM. A third aspect, which is very important for simulating realistic tumors, is the capability of *in silico* cell mutation. Here, cell parameters such as division rate, motility magnitude, cell-cell adhesion etc. must be adjusted at the time of division. All the aforementioned aspects are treated in the macroscale layer. It combines information gathered from the lower layers with higher-level parameters, which results in an agent-based system. Here, the conditions for cell division are checked, the division process is carried out, the motility direction is updated, etc.

By combining micro-, meso- and macroscale, we gain a versatile tool, which can then be parameterized.

### 4.2 Model parameters

As mentioned in section 2.1, we simulated a multitude of spheroids using CiS. We based our simulations on experimental spheroid data provided by our collaborators [13]. Hence, each simulated spheroid had an initial diameter of 200 μm, contained roughly 2000 cells, and was placed in the center of a volume spanning 800 x 800 x 800 μm^3^. Using CiS, we propagated this system at a range of different simulation parameter combinations, which are highlighted below.

#### Extracellular matrix

The extracellular matrix (ECM) is a scaffold within tissues, which connects cells and serves both as a structural component and cell maintenance network [36]. It is composed of overlapping fibrous polymers, such as collagens, proteoglycans and glycoproteins [37]. Tumor spheroids are often placed into a collagen matrix, which serves as a proxy for an *in vivo* ECM [13, 38]. To capture this in our simulations, we modeled the ECM as overlapping, randomly oriented fibers, which were placed within the system volume surrounding the spheroid (see Fig 1a)). These fibers represented rigid obstacles for the cells, to which they could adhere, but which could not be displaced. Since in reality the ECM is not a solid structure, but can be modified and degraded by cells [39], this alone was an overly simplified description. We therefore added a degradation effect, by which cells removed ECM lattice points with which they were in direct contact on the CPM lattice. This occured after a set number of MC steps, as described by the ECM degradation period parameter. During each degradation event, a lattice point in contact with a cell was removed with a probability of 50%. For our simulations, we varied both the ECM density and the ECM degradation period. The ECM density was varied between qualitative values of 729 and 1728 fibers. The ECM degradation was either disabled, or its period was varied between 1 000 and 5 000 MC steps. Finally, we wanted to investigate the effect of ECM alignment. Since it is known that tumors remodel their ECM, and ECM alignment is one of the main drivers of invasion [24], we decided to include both an unaligned and a radially aligned ECM in our studies (see Fig 1a)). We performed simulations both with aligned and unaligned ECM.

#### Cell-cell adhesion

Changes in the adhesion strength between cells are a well known factor which facilitates invasion [7]. We therefore varied the adhesion strength parameter within our simulations, by changing the adhesion coefficient matrix in the third component of Eq 14. This matrix describes the strength of adhesion between different cell types, as well as the strength of adhesion between cells and the ECM.

#### Cell motility magnitude and persistence

Similar to cell-cell adhesion, the cell motility is strongly connected to the invasion properties of cells [40]. To include it, we modified the CPM Hamiltonian by adding a directional potential to each cell with the following contribution [35]:

$$\begin{array}{ccc} H_{\text{CPM},\text{mot}} & =H_{\text{CPM}}+\lambda_{\text{mot}}\cdot \sum_{c\in C}{\vec{m}}_{c}\cdot {\vec{R}}_{c},\; & \text{Cell}\;\text{motility}\\ {\vec{m}}_{c} & =p\cdot ({\vec{R}}_{c}\left(t\right)-{\vec{R}}_{c}\left(t-\Delta t\right))+\left(1-p\right)\cdot \vec{\eta } \end{array}$$ (15)

where ${\vec{m}}_{c}$ is the potential and direction a cell *c* experiences, *R*_c_ is the center of mass of cell *c*, and $\vec{\eta }$ is a random vector obtained by a Wiener process [41]. The motility is implemented as a modified persistent random walk of each cell. The energy contribution of each cell is the dot product of $\vec{R_{c}}$ and $\vec{m_{c}}$, which in turn is determined by the cell’s previous movement and the random vector $\vec{\eta }$. The mixture of persistent and random movement can be chosen by the persistence parameter *p* ∈ [0, 1]. Cells with *p* = 0 perform purely random walks, and cells with *p* = 1 perform purely persistent walks. The coupling strength of this energy term to the CPM is given by λ_mot_.

#### Cell division

Including the effect of cell proliferation is crucial when simulating growing tissues. In CiS, this is implemented as follows: upon division, half of those lattice points on the CPM layer which belong to the dividing cell are assigned a new value, corresponding to the ID of the new cell. The old cell is kept, but its surface, volume, age and generation are adjusted, such that both cells resemble daughter cells of the original one. A division condition needs to be fulfilled in order for a cell to divide. This condition is checked at every MC step. It can be customized, and can include nutrient availability, cell volume, division probability, and cell generation, i.e. the maximum number of divisions per original cell. Since we did not explicitly model nutrient distribution in this study, we focused on the last three aspects. Hence, in our simulations a cell’s volume had to be at least 90% of its target volume. Furthermore, in the experimental data by Kang et al [13], the cell number roughly doubles after three days of growth. Therefore, we set the division probability per MC step such that the overall cell number would double after 250 000 MC steps. Finally, we set the cell generation condition such that each cell could only divide once.

### 4.3 Experimental spheroid preparation and analysis

All experimental methods were previously reported by Kang et al. [13] and are briefly summarized here. Multicellular tumor spheroids were formed by seeding highly invasive, triple-negative MDA-MB-231 breast cancer cells [42] in low-attachment 96 well plates (Corning, No. 07201680) in the presence of 2.5% v/v Matrigel (Corning, No. 354234) [43]. Using this approach, ∼1000 cancer cells coalesced into a spherical aggregate (i.e., a tumor spheroid) of 300-to-400*μ*m in diameter over the course of 48 hours. Once formed, individual spheroids were fully embedded into a 3D fibrous gel prepared using rat-tail collagen I (Corning, No. 354249) [44]. As shown in Kang et al. [13], by varying the collagen concentration between 1 and 4 mg/ml, one can tune the fiber density and overall mechanical properties of the collagen network surrounding each tumor spheroids. MDA-MB-231 spheroids were then cultured in such 3D micro-environments for either 1 hour (Day 0), 24 hours (Day 1), 48 hours (Day 2), or 72 hours (Day 3). While at Day 0 all cells remained within the main spheroid (solid-like phase), over the course of 3 days tumor spheroids progressively developed strikingly different patterns of invasion as a function of collagen concentration, including single cell invasion in 1–2 mg/ml collagen (gas-like phase) and collective invasion in 3–4 mg/ml collagen (liquid-like invasion) [13]. For each time point, spheroids were fixed, optically cleared [45], stained with DAPI (Fisher Scientific, No. D1306), and imaged using a Bruker Ultima Investigator multiphoton microscope equipped with a long working distance 16x water-immersion objective (Nikon, 0.8 N.A., 3mm working distance) to enable whole-spheroid imaging [13]. The 3D positions of DAPI-stained cell nuclei were finally identified using a custom Matlab code developed by Kang et al. [13] and used herein as point cloud data to extract features from experimental spheroids. In this work we used point cloud data from spheroids imaged at days 0–1-2-3 in collagen concentrations of 1–2-3-4 mg/ml, *n* = 3 per group, except for day 0 in 1 mg/ml (*n* = 2), and day 2 in 2 mg/ml (n = 9).

### 4.4 Nastjapy

To facilitate the use of the analysis pipeline presented in this study, we developed the Python package *Nastjapy*, which can be found at http://www.gitlab.com/nastja/nastjapy. For this study, *Nastjapy* served three functions:

1. providing a unified interface for processing data from multiple different sources
2. performing efficient and parallel feature extraction and analysis
3. adaptating and computing the deviation score for specific applications.

We have implemented multiple loading functions into *Nastjapy*, such that it can treat spheroids and other single-cell resolved data from different sources. It currently supports loading point cloud data in the CSV, HDF5, SQLite and matlab file formats. These data are loaded into so-called *DataHandler* objects, which are structured the same way regardless of the origin of the data. Hence, except for the data contained in it, the *DataHandler* object of a simulated spheroid is the same as that of an experimentally measured spheroid. Each *DataHandler* object includes functionalities for visualizing the point cloud data, and extracting the features discussed in section 2.2. Furthermore two or more *DataHandler* objects can be compared with each other. For this, the timesteps to be compared between the spheroids are mapped to each other, and the metric distances are then calculated for each extracted feature. Extracting all features for many different spheroids, possibly at multiple points in time, as well as the comparison between a large number of spheroids, can quickly become computationally expensive. We have therefore added the option to parallelize the feature extraction for individual *DataHandler* objects on multiple CPU cores via MPI. As we discussed in section 2.4, we have implemented a use case adaptation method, in which the feature distances are standardized, and the weight factors for each feature distance are optimized to maximize the relation stated in Eq 7. All functions neccessary to perform this for a set of *DataHandler* objects are implemented in *Nastjapy*.

## Supporting information

S1 Fig Deviation score box plots for simulated phenotypes. **a)** Each phenotype compared to the “deformed” phenotype. **b)** Each phenotype compared to the “spherical with far gaslikes” phenotype. **c)** Each phenotype compared to the “disordered” phenotype. Significance was determined using Welch’s t-test.
(TIF)

S2 Fig Top-down view of spheroid point clouds obtained from *in vitro* MDA-MB-231 spheroids grown in collagen media at four different concentrations *c*. Spheroids were imaged after one, two and three days of growth, and three replicates were imaged per concentration and growth duration. Blue cells are classified as non-gaslike, and red cells are classified as gaslike.
(TIF)

S3 Fig Deviation score box plots of *in vitro* spheroids grown in media at four different collagen concentrations (data provided by Kang et al [13]). The horizontal axis denotes the growth duration of the spheroids within the respective boxplot, and the vertical axis denotes the reference collagen concentration. Significance was determined using Welch’s t-test.
(TIF)

S4 Fig Visualization of the spheroid surface deformation feature on a triangulated spheroid surface. Shown is the surface, composed of many connected triangles, as well as an example of the normal and origin vectors of two triangle vertices. During the feature extraction, the scalar product between these two vectors is calculated for each vertex in the triangle mesh, and grouped in a histogram. Non-deformed surfaces will contain more vertices in which the two vectors are approximately parallel (left example), while strongly deformed surfaces will contain many vertices in which there is a strong deviation between the two (right example).
(TIF)

S5 Fig Sketch of the three layers of Cells in Silico (CiS). A 3D Cellular Potts Model (CPM, shown in 2D for illustrative purpose) at the microscale is combined with nutrient and signal exchange at the mesoscale and an agent-based layer at the macroscale. This enables detailed capture of individual cell dynamics. Adapted from [46].
(TIF)
