# Supplementary figures and images for: Development of a scoring function for comparing simulated and experimental tumor spheroids
Source: PLoS Comput Biol. 2023 Mar 30;19(3):e1010471. doi: 10.1371/journal.pcbi.1010471 (PMC10089329; doi:10.1371/journal.pcbi.1010471)

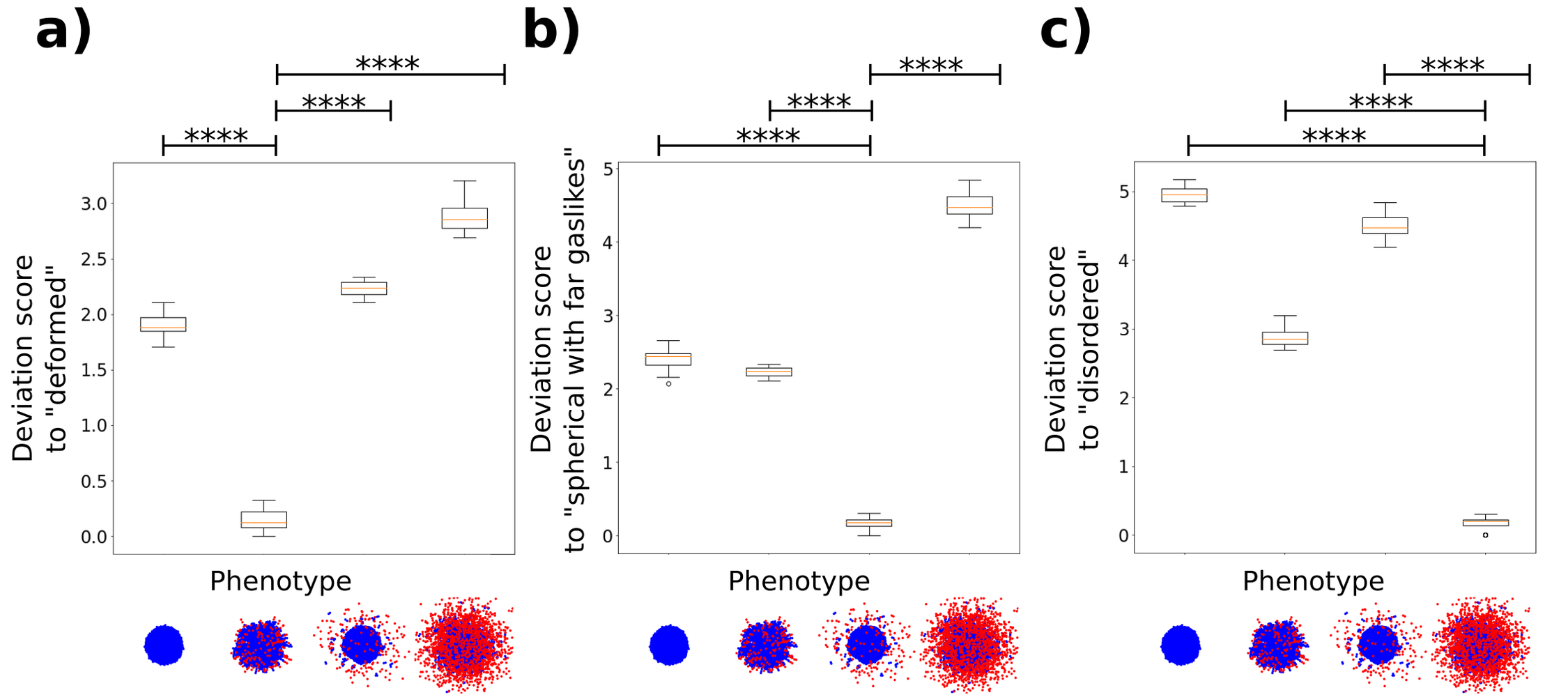

Supplement: S1 Fig — a) Each phenotype compared to the “deformed” phenotype. b) Each phenotype compared to the “spherical with far gaslikes” phenotype. c) Each phenotype compared to the “disordered” phenotype. Significance was determined using Welch’s t-test. (TIF) [file pcbi.1010471.s001.tif]

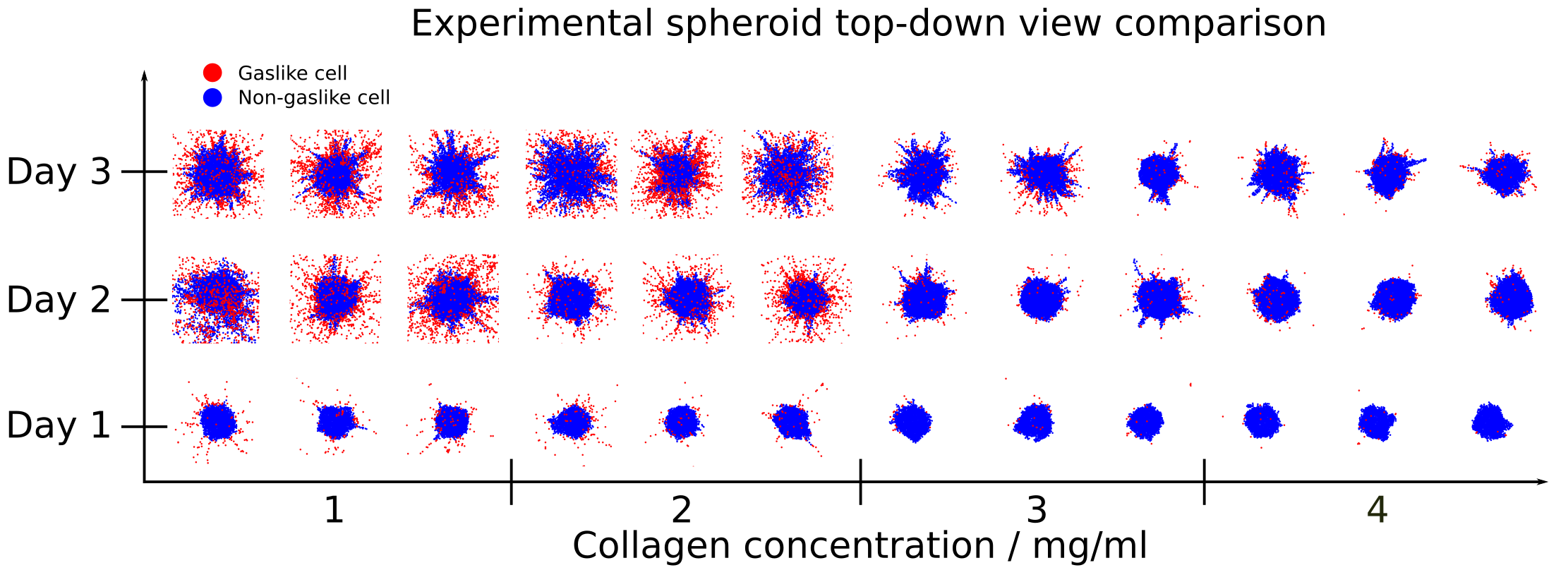

Supplement: S2 Fig — Spheroids were imaged after one, two and three days of growth, and three replicates were imaged per concentration and growth duration. Blue cells are classified as non-gaslike, and red cells are classified as gaslike. (TIF) [file pcbi.1010471.s002.tif]

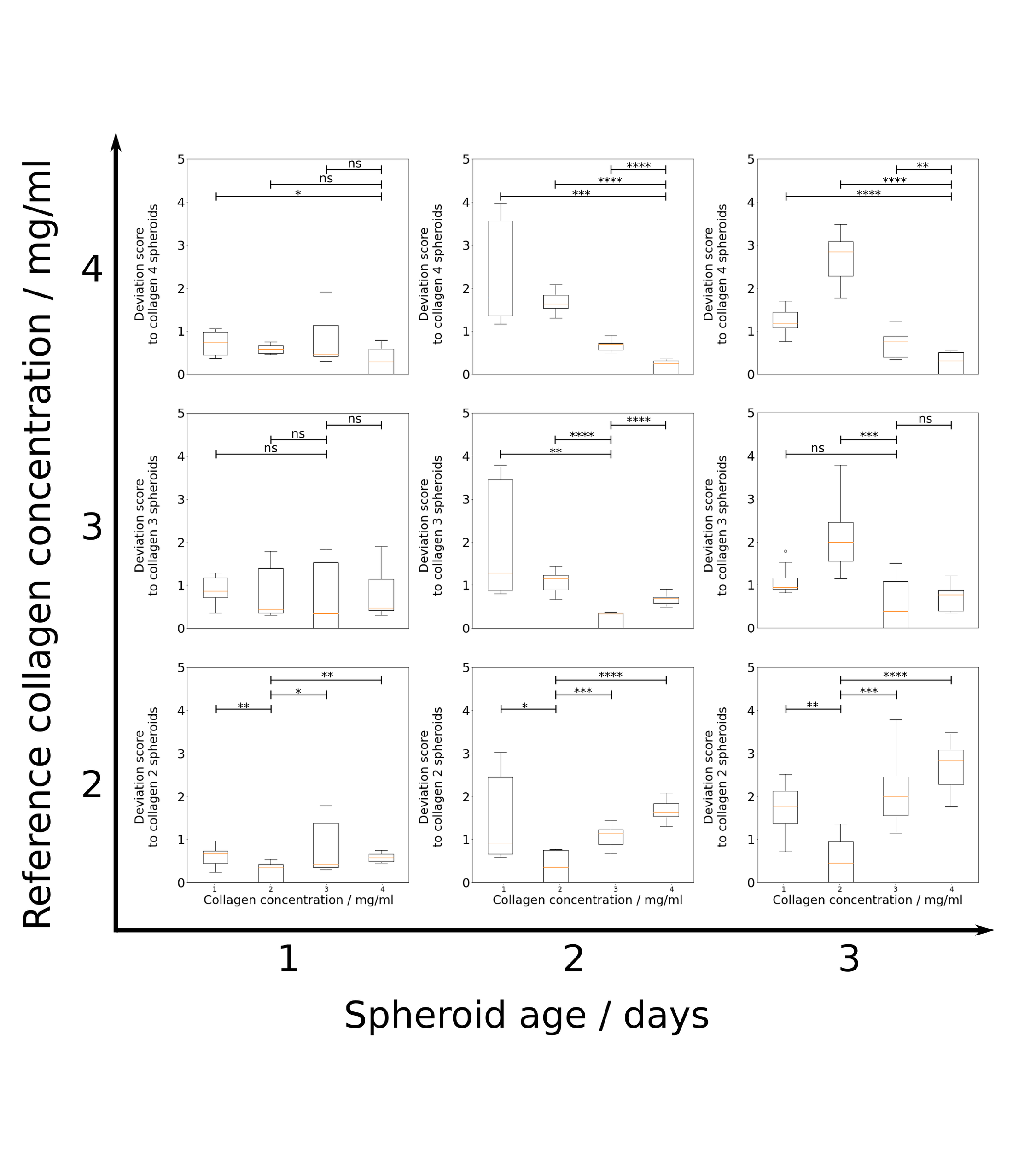

Supplement: S3 Fig — The horizontal axis denotes the growth duration of the spheroids within the respective boxplot, and the vertical axis denotes the reference collagen concentration. Significance was determined using Welch’s t-test. (TIF) [file pcbi.1010471.s003.tif]

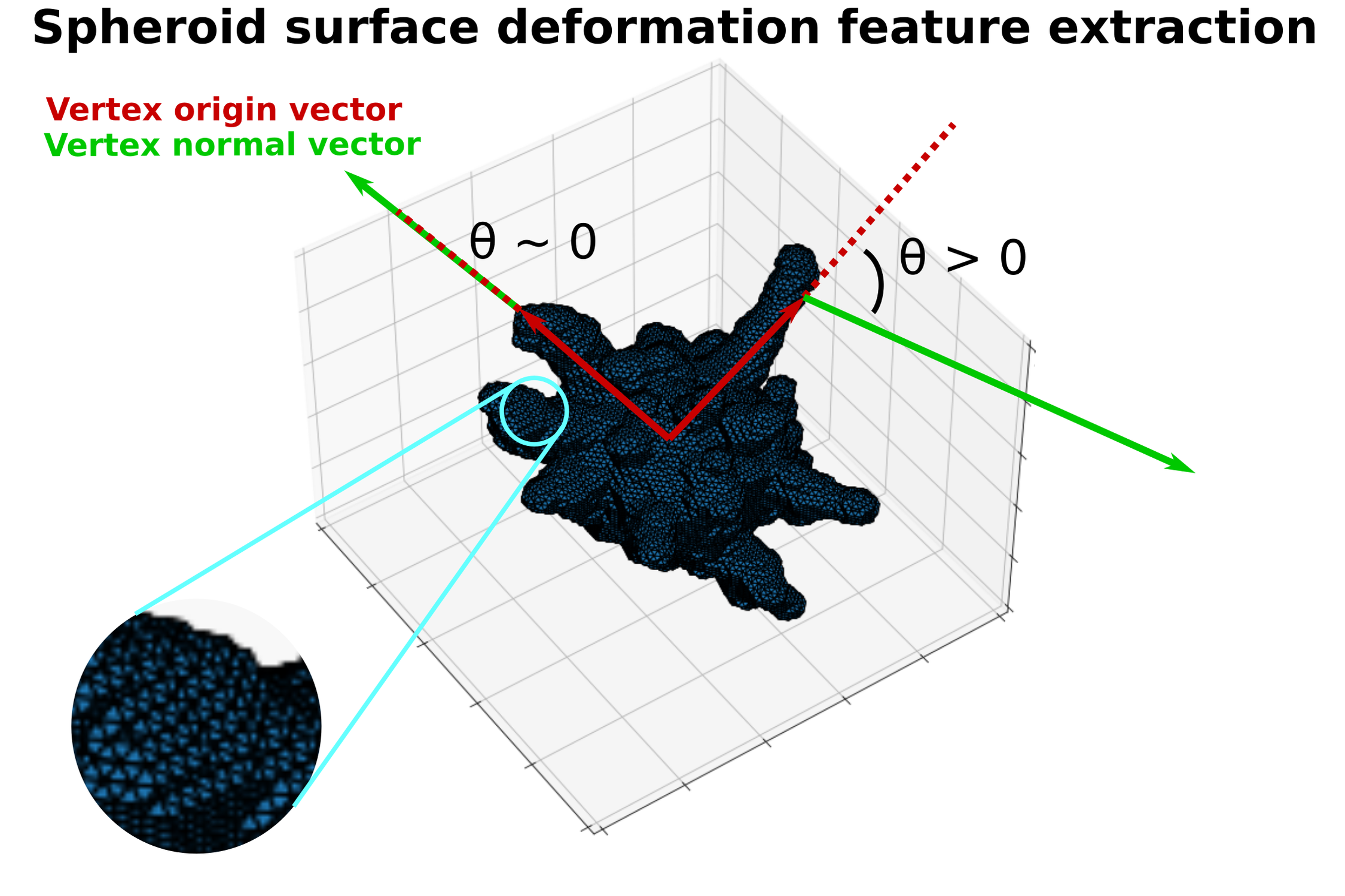

Supplement: S4 Fig — Shown is the surface, composed of many connected triangles, as well as an example of the normal and origin vectors of two triangle vertices. During the feature extraction, the scalar product between these two vectors is calculated for each vertex in the triangle mesh, and grouped in a histogram. Non-deformed surfaces will contain more vertices in which the two vectors are approximately parallel (left example), while strongly deformed surfaces will contain many vertices in which there is a strong deviation between the two (right example). (TIF) [file pcbi.1010471.s004.tif]

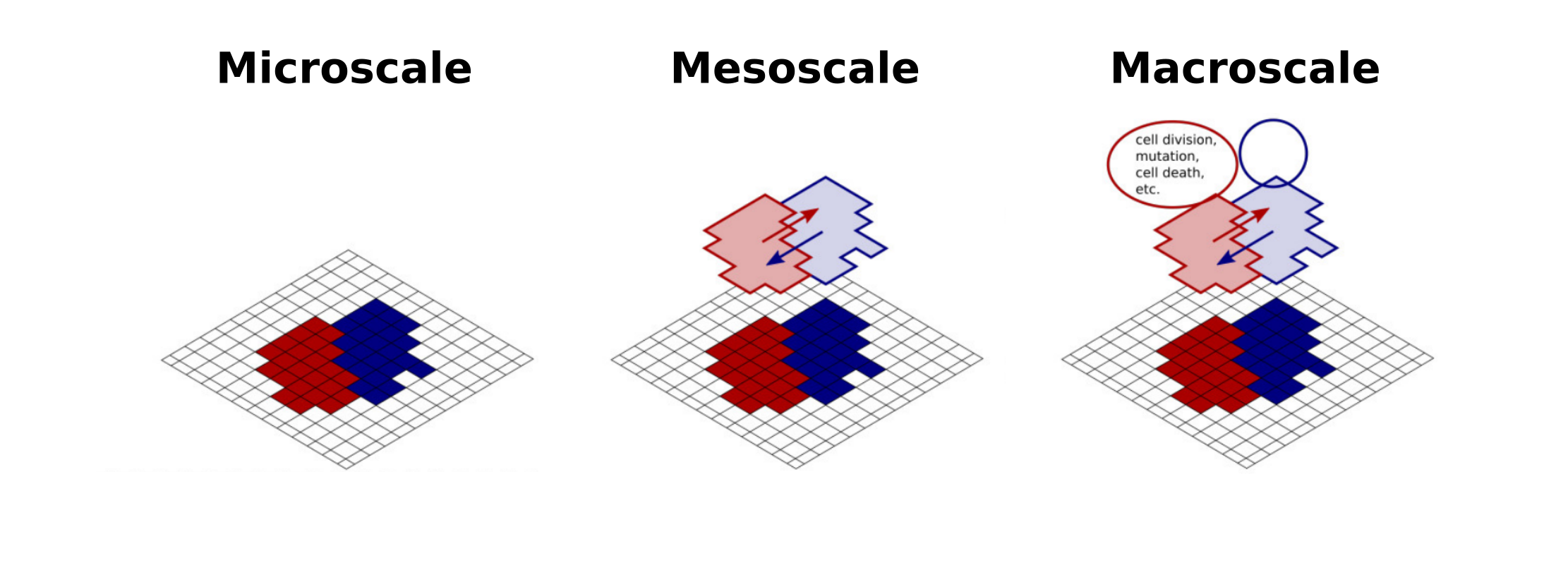

Supplement: S5 Fig — A 3D Cellular Potts Model (CPM, shown in 2D for illustrative purpose) at the microscale is combined with nutrient and signal exchange at the mesoscale and an agent-based layer at the macroscale. This enables detailed capture of individual cell dynamics. Adapted from [46]. (TIF) [file pcbi.1010471.s005.tif]
